# Representational learning by optimization of neural manifolds in an olfactory memory network

**DOI:** 10.1101/2024.11.17.623906

**Published:** 2024-11-18

**Authors:** Bo Hu, Nesibe Z. Temiz, Chi-Ning Chou, Peter Rupprecht, Claire Meissner-Bernard, Benjamin Titze, SueYeon Chung, Rainer W. Friedrich

**Affiliations:** 1 Friedrich Miescher Institute for Biomedical Research, Fabrikstrasse 24, 4056 Basel, Switzerland; 2 University of Basel, 4003 Basel, Switzerland; 3 Center for Computational Neuroscience, Flatiron Institute, New York, NY, USA; 4 Neuroscience Center Zurich, 8057 Zurich, Switzerland; 5 Brain Research Institute, University of Zurich, 8057 Zurich, Switzerland; 6 Center for Neural Science, New York University, New York, NY, USA

## Abstract

Higher brain functions depend on experience-dependent representations of relevant information that may be organized by attractor dynamics or by geometrical modifications of continuous “neural manifolds”. To explore these scenarios we analyzed odor-evoked activity in telencephalic area pDp of juvenile and adult zebrafish, the homolog of piriform cortex. No obvious signatures of attractor dynamics were detected. Rather, olfactory discrimination training selectively enhanced the separation of neural manifolds representing task-relevant odors from other representations, consistent with predictions of autoassociative network models endowed with precise synaptic balance. Analytical approaches using the framework of *manifold capacity* revealed multiple geometrical modifications of representational manifolds that supported the classification of task-relevant sensory information. Manifold capacity predicted odor discrimination across individuals, indicating a close link between manifold geometry and behavior. Hence, pDp and possibly related recurrent networks store information in the geometry of representational manifolds, resulting in joint sensory and semantic maps that may support distributed learning processes.

## INTRODUCTION

Learning generates organized representations of relevant information in the brain that generalize to novel inputs and serve as a basis for cognition. Representational learning is thought to depend on autoassociative memory networks that store information by activity-dependent modifications of recurrent synaptic connectivity. Classical models predict that learning enhances recurrent excitation among specific ensembles of neurons, resulting in convergent attractor dynamics that mediates pattern classification^1-5^. A recurrent network can also give rise to a continuum of stable attractor states that may, for example, function as a cognitive map^2,6^. Alternatively, representational learning may be mediated by mechanisms that do not depend on attractor dynamics. Generally, networks may represent relevant information by mapping patterns of input activity to specific subspaces of neuronal state space according to semantic relationships between inputs. Hence, learning may cause geometrical state space modifications that define continuous *neural manifolds*, rather than distinct attractor states^7-14^ but the possible functions of such manifolds in autoassociative memory remain to be explored.

Autoassociative memory has been proposed to be a primary function of piriform cortex, a paleocortical brain area that receives non-topographic sensory input from the olfactory bulb. Within piriform cortex, excitatory neurons are recurrently connected through an extensive “association fiber system” that is plastic and under neuromodulatory control^15-17^. These observations gave rise to the long-standing hypothesis that learning results in the formation of neuronal assemblies in piriform cortex that represent odor objects and mediate pattern classification by convergent dynamics^18,19^. However, recent analyses of neuronal activity in piriform cortex did not reveal obvious signatures of attractor states. For example, odor-evoked activity is not persistent but phasic and curtailed by inhibition^20-22^. Moreover, while convergent dynamics are expected to reduce variability, intra- and inter-trial variability of neuronal activity in piriform cortex appears high in comparison to inputs from the olfactory bulb^22-25^. Nonetheless, passive odor exposure and active learning modify odor-evoked population activity in piriform cortex depending on stimulus statistics or task structure^24,26-29^. For example, activity patterns evoked by different odors become more highly correlated after presentation of odors in a binary mixture^24^, or after association of multiple odors with a common reward^28^. Hence, experience may reorganize odor representations in piriform cortex in a task-relevant fashion by mechanisms that do rely on discrete attractor dynamics.

Autoassociative memory has recently been explored in computational models of recurrent networks that were constrained by data from telencephalic area pDp of adult zebrafish^30^, the teleost homolog of the piriform cortex^31,32^. When information was stored in these models by enhancing connectivity among small assemblies of excitatory and inhibitory neurons, a transition from discrete attractor dynamics to continuous representational manifolds occurred when networks entered a regime of precise synaptic balance^30^. This regime is characterized by strong and correlated excitatory and inhibitory synaptic currents in individual neurons and thought to be characteristic of cortical circuits^33,34^. As manifolds may be constrained primarily in specific state space dimensions the retrieval of information may depend on the selection of neuronal subsets for readout^30^.

As neural manifolds in the brain may be geometrically complex and embedded in a high-dimensional state space it is not trivial to describe their geometry and to determine the impact of manifold geometry on neuronal computations. A prerequisite for the analysis of neural manifolds in biological datasets are simultaneous measurements of activity across large neuronal populations. Basic features of manifolds may be assessed using two distance measures, Euclidean and Mahalanobis distance, that together provide information about the relative separation and orientation of manifolds. Moreover, geometrical features of neural manifolds relevant for pattern classification can be analyzed using the mathematical framework of *manifold capacity*, a measure that quantifies the degree of separability - or untangleness - of manifolds as a function of geometrical parameters^9,14,35^. Recently, this framework has been extended into GCMC (Geometric measures from Correlated Manifold Capacity) theory, which also considers the impact of multiple forms of neuronal co-variability on manifold capacity^36^. Thus, manifold capacity quantifies the amount of linearly decodable information per neuron as a function of parameters describing manifold geometry and correlations. Recently, GCMC theory has been used successfully to quantify computationally relevant, interpretable features of neuronal population activity in different brain areas and tasks^36^. Manifold capacity analysis can therefore be used to determine how learning modifies geometrical features of representational manifolds, and how these modifications collectively affect the classification of activity representing learned and novel inputs.

To explore how learning modifies representational manifolds we measured population activity in pDp after training juvenile or adult zebrafish in an odor discrimination task. Previous studies showed that odor-evoked activity in pDp is distributed, variable, modified by experience, and under strong inhibitory control^37-41^, consistent with observations in piriform cortex^18-20,22-24,28,29,42,43^. Moreover, in both brain areas, synaptic currents are dominated by recurrent rather than afferent input during an odor response^17,38,40,44^, indicating that they function as autoassociative networks. In pDp, voltage clamp recordings demonstrated that excitatory and inhibitory synaptic currents in individual neurons are strong and co-tuned, providing direct evidence for precise synaptic balance^40^. The analysis of neuronal population activity in pDp therefore allowed us to examine how learning modifies representational manifolds and to test predictions of computational models^30^.

Consistent with various previous observations we found no obvious signatures of fixed-point attractor dynamics in pDp. Behavioral odor discrimination training had only minor effects on the global amplitude and variability of odor responses in pDp. Nonetheless, training modified activity manifolds representing learned and related odors, with minimal effects on representations of dissimilar odors. Training modified multiple geometrical features that, in combination, enhanced the separability of manifolds representing task-relevant odors from each other and from manifolds representing novel odors (Supplementary Fig. S1). The separability of activity patterns was correlated with behavioral odor discrimination across trained individuals, indicating that representational manifolds directly contribute to learned behaviors. These results indicate that pDp stores task-relevant information not as discrete items but in continuous neural manifolds that improve classification of sensory information along relevant directions in coding space. Hence, representational learning in pDp, and possibly in other recurrent balanced-state networks, integrates sensory and semantic information into a continuous map, which may support distributed learning processes.

## RESULTS

### Basic features of odor-evoked neural dynamics in pDp

**Fig. 1 ∣:**
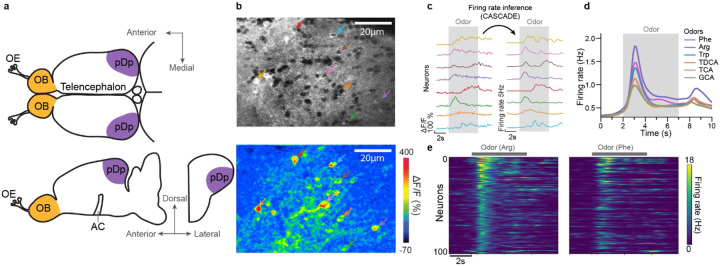
Two-photon Ca^2+^ imaging of odor-evoked activity in pDp of juvenile zebrafish **a,** Location of pDp in the telencephalon of juvenile zebrafish. OB: olfactory bulb. **b,** Top: αTubulin:GCaMP6s expression in juvenile pDp (2-photon optical section; subregion of entire field of view). Relative change in fluorescence (ΔF/F) evoked by odor stimulation (Arg) in the same field of view, averaged over 3 s. **c,** Left: ΔF/F traces of neurons depicted by arrows with matching colors in **b**. Right: Firing rate inferred from ΔF/F traces by CASCADE. Gray bar indicates odor application. **d,** Inferred spike probability (firing rate) as a function of time, averaged over all neurons (n = 9059) and fish (n = 6; naïve juvenile group). Gray shaded area indicates odor application. **e,** Inferred firing rate of 100 randomly selected Dp neurons from juvenile fish before, during and after stimulation with Arg (left) and Phe (right) odors, sorted by mean response intensity to Arg. Gray bar indicates odor application.

We measured odor-evoked activity in an ex-vivo preparation of the juvenile and adult zebrafish brain by volumetric 2-photon calcium imaging^45,46^ in transgenic fish expressing the calcium indicator GCaMP6s (Tg[alphaTubulin:GCaMP6s]) throughout the telencephalon and other brain areas^47^ (Fig. 1a,b). 3D scanning was performed by remote focusing with a volume rate of 7.5 Hz and calcium signals (ΔF/F) were transformed into firing rate estimates using CASCADE (Fig. 1c)^41^. Activity across populations of neurons was represented by n-dimensional activity vectors, each representing the estimated firing rates of n neurons within a time bin. Most experiments were performed in juvenile fish (46 – 56 days post fertilization; 9.0 – 13.5 mm body length), which allowed us to measure activity across the majority of neurons in pDp. Consistent with previous results from adult zebrafish^37-40^, odor-evoked activity in juvenile pDp exhibited an initial phasic increase followed by a slowly decaying plateau (Fig. 1d,e), and responses of individual neurons were scattered throughout pDp without an obvious odor-related topography.

**Fig. 2 ∣:**
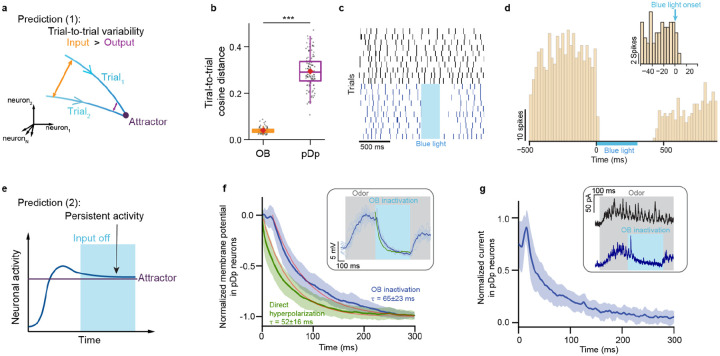
Testing for signatures of fixed-point attractor networks **a,** Schematic illustration of noise reduction by convergent attractor dynamics. **b,** Trial-to-trial variability of odor-evoked firing rates in the olfactory bulb (OB) and pDp of the same fish (n = 6;). Trial-to-trial variability was quantified by the cosine distance between activity patterns averaged over a 3 s time window during odor application in each trial. Variability was higher in pDp (mean ± SD 0.29 ± 0.07, n_trial_pair_ = 3 trial pairs, n_odor_ = 6 odors from N = 6 fish) than in the olfactory bulb (0.04 ± 0.01; Mann–Whitney U test, U = 11664.00, P = 6.1 × 10^−37^), contradicting attractor-based predictions (**a**). **c,** Action potentials of a mitral cell in different trials (rows) during odor application without (black) and with (blue) optogenetic stimulation of inhibitory interneurons in Tg[dlx4/6:ChR2-YFP] fish. Cyan bar indicates blue light stimulus (300 ms). **d,** Peri-stimulus time histogram (PSTH; 4 mitral cells from two fish, 109 trials, 20 ms bins) of spiking activity around the time of optical stimulation (blue bar). Inset shows a zoom-in (5 ms bins). **e,** Schematic illustration of persistent activity during an attractor state. **f,** Mean membrane potential change in pDp neurons after optogenetic silencing of the olfactory bulb (blue) and upon injection of a hyperpolarizing step current (green, 500 ms; n = 17 pDp neurons from 6 fish, in total 177 trials; shaded area shows SD). Individual traces were normalized prior to averaging. Red lines show single-exponential fits averaged over neurons; numbers show mean time constants (± SD). The first 20 ms was excluded for fits to traces with optical stimulation to account for delayed silencing of olfactory bulb output. Inset shows membrane potential time course of a single neuron during odor stimulation (gray shading) and optogenetic silencing of olfactory bulb output (cyan shading). Thin traces show individual trials (n_trial_ = 9); dark blue trace shows average. The gray line shows the average membrane potential change in response to step current injection (first 300 ms). **g,** Mean normalized excitatory postsynaptic currents (EPSCs) during an odor response after optogenetic silencing of olfactory bulb output (n = 9 pDp neurons from 4 fish, total of 54 trials; voltage clamp recordings). Traces from individual neurons were normalized prior to averaging. Inset shows EPSCs evoked by the same odor in the same neuron in single trials without (top) or with (bottom) optogenetic silencing of olfactory bulb output (cyan shading).

Previous results from pDp and piriform cortex failed to provide strong evidence for convergent attractor dynamics in response to novel or learned odors^21-25,29,37-40,42^. To confirm these observations, we examined two signatures of attractor states: (1) as a consequence of convergent dynamics, trial-to-trial variability is expected to be lower in output activity patterns than in input patterns (Fig. 2a), and (2) because attractor states are stable, activity is expected to persist for some time when input is switched off abruptly (Fig. 2e).

To test the first prediction, we measured activity evoked by a panel of odors in pDp and in the olfactory bulb of the same juvenile zebrafish. Trial-to-trial variability was quantified as the cosine distance between activity vectors evoked by the same odors in different trials, averaged over a 3 s time window starting 1 s after response onset. Consistent with previous observations^37,38,48-50^, variability was high in pDp but low in the olfactory bulb (Fig. 2b), contradicting expectations for attractor networks.

To test the second prediction, we used Tg[dlx4/6:Chr2YFP] zebrafish, which express channelrhodopsin-2 fused to YFP (Chr2YFP) in superficial inhibitory interneurons of the olfactory bulb^51,52^. In these fish, olfactory bulb output could be silenced by targeted full-field illumination with blue light within 10-20 ms (Fig. 2c,d). We then performed whole-cell current- and voltage-clamp recordings from individual pDp neurons to examine the evolution of network activity after rapid silencing of olfactory bulb output during an odor response. Consistent with previous observations^38,40^, odor stimulation evoked a barrage of oscillatory excitatory synaptic currents and a depolarization that occasionally resulted in action potential firing of pDp neurons. Silencing of olfactory bulb output during an odor response resulted in a fast hyperpolarization of pDp neurons with a time constant of 65 ± 23 ms, as determined by exponential fits. Direct injection of a step current hyperpolarized the same pDp neurons with a time constant of 52 ± 16 ms (Fig. 2f). Excitatory synaptic currents measured by voltage clamp recordings decayed with a similar time course (Fig. 2g). Hence, pDp did not exhibit obvious signs of persistent activity. Rather, network activity decayed after silencing of sensory input on a timescale similar to the intrinsic time constant of pDp neurons. Together, these observations support the notion that odor-evoked activity in pDp does not show obvious signs of fixed-point attractor dynamics.

### Experience-dependent modification of neural dynamics in pDp

**Fig. 3 ∣:**
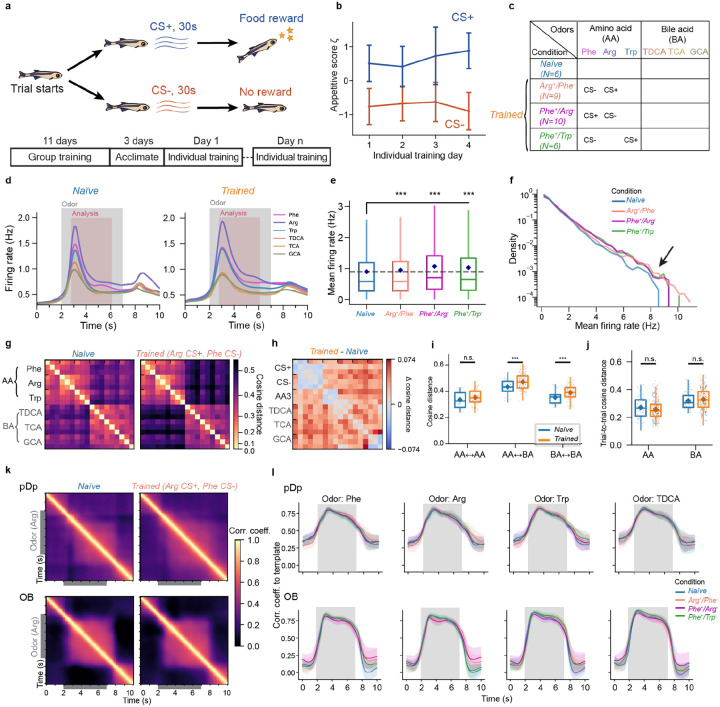
Effects of odor discrimination training on neural dynamics in pDp: basic observations **a,** Schematic of odor discrimination task for juvenile fish. Fish were trained as a group in a home tank (11 days), acclimated to a single tank (3 days), and further trained individually for 2 - 4 days. Behavior was quantified during individual training. **b,** Appetitive scores ζ (mean ± SD) for ${\text{CS}}^{+}$ and ${\text{CS}}^{-}$ odors on each day of individual training from all trained fish. The appetitive scores were significantly higher for ${\text{CS}}^{+}$ than ${\text{CS}}^{-}$ on all days (day 1, n = 25, P = 6.0 × 10^−8^; day 2, n = 25, P = 1.8 × 10^−7^; day 3, n = 21, P = 6.7 × 10^−6^; day 4, n = 14, P = 1.2 × 10^−4^; Wilcoxon signed-rank test). **c,** Odor panel and reward assignments in different training groups. **d,** Mean inferred firing rate evoked by each odor, averaged across all neurons and trials in fish (N = 6 fish, n = 9059 neurons; left; same as in Fig. 1d) and in trained fish (N = 25 fish, n = 34152 neurons; all training groups combined; right). Gray shaded area indicates odor application. Red shaded area represents the 3 s time window used for analysis of response amplitudes, pattern cosine distances and manifold distances. **e,** Mean activity evoked by amino acid odors in different training groups. Activity was significantly higher in all trained groups (Arg^+^/Phe^−^: mean ± SD 0.95 ± 1.06, n_odor_ = 3, n = 13013 neurons from N = 9 fish; Phe^+^/Arg^−^: 1.07 ± 1.10, n_odor_ = 3, n = 13379 neurons from N = 10 fish); Phe^+^/Trp^−^: 1.03 ± 1.11, n_odor_ = 3, n = 7760 neurons from N = 6 fish) than in naïve fish (0.90 ± 0.93, n_odor_ = 3, n = 9059 neurons from N = 6 fish; Kruskal–Wallis test, n = 129633, P = 3.7 × 10^−143^). Posthoc Dunn test: Arg^+^/Phe^−^ vs naïve, P = 2.9 × 10^−10^; Phe^+^/Arg^−^ vs naïve, P = 6.1 × 10^−104^; Phe^+^/Trp^−^ vs naïve, P = 2.8 × 10^−45^. **f,** Distribution of firing rates evoked by amino acid odors in different training groups. High firing rates were more frequently observed in trained fish (arrow). **g,** Mean cosine distance between activity evoked by different odors (3 trials each) in naïve fish (left) and in fish trained on Arg as ${\text{CS}}^{+}$ and Phe as ${\text{CS}}^{-}$ (Arg^+^/Phe^−^). **h,** Mean difference between distance matrices from trained and naïve fish. Amino acid odors were reordered by reward assignment (${\text{CS}}^{+}$, ${\text{CS}}^{-}$, third amino acid) for each training group. **i,** Cosine distances between trial-averaged activity vectors representing different odor categories in naïve and trained fish. Amino acids vs amino acids (AA-AA), naïve (mean ± SD): 0.336 ± 0.064, n = 18 odor pairs from N = 6 fish; trained: 0.354 ± 0.053, n = 75 odor pairs from N = 25 fish, Mann–Whitney U test, P = 0.107. Amino acids vs bile acids (AA-BA), naïve: 0.433 ± 0.058, n = 54 odor pairs from N = 6 fish,; trained: 0.471 ± 0.057, n = 225 odor pairs from N = 25 fish, Mann–Whitney U test, P = 0.0002. Bile acids vs bile acids (BA-BA), naïve: 0.354 ± 0.055, n = 18 odor pairs from N = 6 fish; trained: 0.389 ± 0.054, n = 75 odor pairs from N = 25 fish, Mann–Whitney U test, P = 0.0007. **j,** Cosine distance between activity vectors of different trials (trial-to-trial variability), analyzed separately for amino acids (AA) and bile acids (BA) in naïve and trained fish. Amino acids, naïve (mean ± SD): 0.270 ± 0.076, n = 54 trial pairs from N = 6 fish, [trained] 0.257 ± 0.069, n = 225 trial pairs from N = 25 fish, Mann–Whitney U test, P = 0.202. Bile acids, naïve: 0.318 ± 0.060, n = 54 trial pairs from N = 6 fish; trained: 0.329 ± 0.076, n = 225 trial pairs from N = 25 fish, Mann–Whitney U test, P = 0.264). **k,** Pearson correlation between activity patterns at different time points in pDp (top) and the OB (bottom), averaged over all trials with the same odor stimulus (Arg) and all fish of the naïve (left) and the Arg^+^/Phe^−^ training group (right). Purple bars indicate odor application; gray bars indicate time window for computation of time-averaged template pattern. **l,** Pearson correlation between activity patterns at each time point and the template pattern in pDp (top; averaged over time indicated by gray shading) and the olfactory bulb (bottom), averaged over trials and fish. Curves show time courses of correlations to the template for responses to four odor stimuli (Phe, Arg, and Trp and TDCA) in each training group (colors; mean ± SD).

To explore how odor representations in pDp are modified by learning we trained juvenile zebrafish expressing GCaMP6s^53^ in the telencephalon in an odor discrimination task using procedures developed for adult fish^54^ with minor modifications. During training, tanks were continuously perfused with fish water and two amino acid odors (${\text{CS}}^{+}$, ${\text{CS}}^{-}$) were each added to the perfusion 9 times per day for 30 s. Presentation of the ${\text{CS}}^{+}$, but not the ${\text{CS}}^{-}$, was followed immediately by food delivery at a specific location. Fish were initially pre-trained as a group for 11 days before individual fish were further trained for 2 to 4 days in separate tanks (Methods; Fig. 3a). Learning was monitored by quantifying appetitive behavior of individual fish during odor presentation as described^54^. After training, appetitive behavior was significantly more pronounced in response to the ${\text{CS}}^{+}$ than to the ${\text{CS}}^{-}$ (Fig. 3b), as observed in adult fish (Namekawa et al., 2018; Frank et al., 2019).

One group of juvenile fish (Arg^+^/Phe^−^; N = 9 fish) was trained using arginine (Arg) as ${\text{CS}}^{+}$ and phenylalanine (Phe) as ${\text{CS}}^{-}$ while a second group (Phe^+^/Arg^−^; N = 10 fish) was trained on the opposite association. A third group (Phe^+^/Trp^−^; N = 6 fish) was trained on Phe as ${\text{CS}}^{+}$ and tryptophan (Trp) as ${\text{CS}}^{-}$ (Fig. 3c). After behavioral training, odor-evoked activity was measured in pDp of all fish and compared to odor responses in a control group of naïve fish (N = 6). The odor panel comprised three amino acids (Phe, Arg, Trp) and three bile acids (TDCA, TCA, GCA), representing two classes of natural odors for aquatic animals^55^. In the olfactory bulb, activity patterns evoked by these odorants were modestly correlated within each chemical class but uncorrelated across classes^37^. Each stimulus was applied three times for 5 s.

As observed in adult pDp^37-40^, mean odor responses in juvenile pDp followed a phasic-tonic time course, with tonic components being somewhat more pronounced in trained fish (Fig. 3d). Mean odor responses to amino acids were slightly but significantly larger in each of the trained groups as compared to naïve fish (Fig. 3e), which could be attributed in part to an overrepresentation of large odor responses in a subset of individual neurons (Fig. 3f). The cosine distance between time-averaged activity patterns evoked by the same odor in different trials was only slightly lower than the cosine distance between activity patterns evoked by different odors from the same odor category. In trained fish, cosine distances between activity patterns evoked by the ${\text{CS}}^{+}$ and the ${\text{CS}}^{-}$ were similar to the corresponding distances in naïve fish, while other cosine distances were slightly higher (Fig. 3g-j). Hence, training modified relationships between odor-evoked activity patterns but response variability remained high.

While high variability is atypical for fixed-point attractors it does not exclude chaotic attractor states. Modeling studies showed that chaotic attractor states tend to result in persistent pattern correlations, even when variability is high^4^. However, we found that correlations between activity patterns at different time points dropped rapidly after stimulus offset, without an obvious difference between naïve and trained animals (Fig. 3k,l). Hence, our observations are not consistent with the hypothesis that training establishes convergent attractor dynamics representing specific odors.

### Basic analysis of manifold geometry in a computational model

**Fig. 4 ∣:**
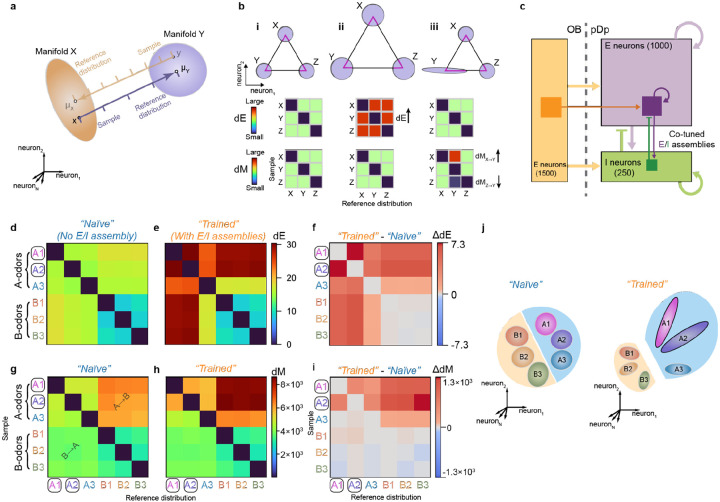
Effects of learning on distances between manifolds: predictions of a spiking network model **a,** Mahalanobis distance: schematic illustration. Yellow and purple ellipsoids depict neural manifolds (sets of activity vectors evoked by an odor, pooled over trials and time points). The Mahalanobis distance from a sample point x in manifold X to the reference distribution computed from manifold Y is the distance from x to the center $\mu_{\text{Y}}$ of Y, relative to the directional variability in Y. ${\text{dM}}_{\text{X}\to \text{Y}}$ is the mean Mahalanobis distance from each point in manifold X to manifold Y. Note that dM is usually asymmetric because the variability in the reference distributions (manifolds) is usually unequal. **b,** Example sets of three manifolds (X, Y, Z) illustrating effects of linear scaling (ii) and geometrical modifications (iii) on dE (top) and dM (bottom). **c,** Schematic of the spiking neural network model. 1000 recurrently connected, excitatory pDp neurons (E; purple) and 250 recurrently connected inhibitory neurons (I; green) received input from 1500 excitatory mitral cells (olfactory bulb; yellow). Information about defined odors (“memory”) was stored in the recurrent connectivity matrix (“learning”) by increasing the connection probabilities among strongly activated E and I neurons (“E/I assembly”). **d,** Matrix showing dE between manifolds representing six odors (input patterns) in a network without E/I assemblies. Odors were separated into two classes (A and B) by their correlations, mimicking biological observations. **e,** Matrix showing dE between representations of the same odors in a network with E/I assemblies representing odors A1 and A2 (“learned” odors). **f,** Difference between dE matrices of networks with and without E/I assemblies representing A1 and A2. Note that E/I assemblies (“learning”) increased dE between learned and other odors, and that this effect generalized to a related odor (A3). **g-i,** Equivalent matrices of dM. E/I assemblies (“learning”) increased dM selectively in the direction from learned to other odors (${\text{dM}}_{\text{A1}\to \text{other}}$; ${\text{dM}}_{\text{A2}\to \text{other}}$), and this effect also generalized to the related odor (${\text{dM}}_{\text{A3}\to \text{other}}$). **j,** Intuitive illustration of the consequences of learning on manifold geometry. E/I assemblies for learned odors A1, A2 result in a directional displacement and extension of the corresponding manifolds that can be attributed, at least in part, to a modest amplification of activity within the assembly. This effect generalizes to the manifold representing a related odor (A3). The odor-specific changes indE (**f**) and dM (**i**) were used as predictions for experimental analyses.

We next analyzed odor-evoked population activity in pDp as neural manifolds. To characterize the geometry of representational manifolds we first analyzed two distance measures, Euclidean distance (dE) and Mahalanobis distance (dM), in neuronal state space (where each dimension represents activity of one neuron). dE was defined as the distance between the centers of two distributions (e.g., the mean of data points representing a manifold). Hence, dE quantifies the separation of prototypical odor representations in state space. dM further depends on the shape of distributions. dM measures the distance between a point (e.g., an individual activity vector) and a reference distribution (e.g., a neural manifold) in units that depend on the shape and extent (covariance pattern) of the reference distribution. dM between two manifolds is thus computed by averaging over the dM between each datapoint in one manifold and the distribution representing the other manifold. Assuming that manifolds have different shapes, dM in the direction from manifold A to manifold B (${\text{dM}}_{\text{A}\to \text{B}}$) is usually different from dM in the opposite direction (${\text{dM}}_{\text{B}\to \text{A}}$), providing information about manifold geometry (Fig. 4a). For example, ${\text{dM}}_{\text{A}\to \text{B}}$ > ${\text{dM}}_{\text{B}\to \text{A}}$ implies that A is more elongated than B along the A-B axis. Because dM is normalized by covariance it is closely related to the separability of two distributions.

We define a manifold representing a given odor as the distribution in state space of all activity vectors measured in response to the odor, pooled over trials. To determine dE between two manifolds, we averaged activity patterns evoked by the same odor at different time points during odor presentation and in different trials, resulting in a single point in state space (“manifold center”). dE between representations of different odors was then determined as the Euclidean distance between manifold centers. Hence, dE is symmetric for any given odor pair and depends both on the angular separation of manifold centers and their amplitudes (firing rates).

dM was determined between individual activity vectors x (activity evoked by an odor in an individual trial and time bin) and activity distributions Y (the set activity vectors evoked by another odor in multiple trials and time bins). dM is defined as

$$dM(x,Y)=\sqrt{{\left(x-\mu_{Y}\right)}^{T}{S_{Y}}^{-1}(x-\mu_{Y})}$$

where $\mu_{Y}$ and $S_{Y}$ are the mean and the covariance matrix, respectively, of distribution Y. Hence, dM is the effective distance between x and the mean of Y, taking into account the variability in Y. dM between manifolds representing odors X and Y in the direction X→Y (${\text{dM}}_{X\to Y}$) was calculated by averaging over dM between each vector x in X and manifold Y. The same procedure was used to determine dM in the opposite direction (${\text{dM}}_{Y\to X}$). Because ${\text{dM}}_{X\to Y}$ is usually not equal to ${\text{dM}}_{Y\to X}$ (Fig. 4a) matrices quantifying dM between multiple manifolds contain information about their separation and geometry along selected directions.

We then jointly quantified dE and dM for manifolds representing different odors to obtain basic insights into their separation and geometry. As illustrated in Fig. 4bi, all dE and dM are equal when manifolds are equidistant and spherical with equal variance. When manifolds are scaled uniformly, dE changes while dM remains constant because Euclidean distance and variance co-scale (Fig. 4bii). When only the geometry of one manifold is modified, dE is unaffected but dM changes between specific subsets of manifolds (Fig. 4biii). Modifying both Euclidean distance and geometry will thus result in specific patterns of changes in dE and dM. Matrices of dE and dM therefore contain basic information about distances between representational manifolds and about their geometry in a subset of state space dimensions (between manifold centers).

To predict effects of learning on dE and dM we simulated odor-evoked activity patterns using recurrently connected networks of spiking neurons (Fig. 4c). Previous models of adult pDp^30^ consisted of 4000 excitatory and 1000 inhibitory neurons. Learning was assumed to enhance connectivity between small assemblies of strongly activated neurons (100 excitatory and 25 inhibitory neurons), which resulted in the separation of manifolds representing learned and related odors from other manifolds. To adapt these computational models to juvenile fish we reduced network size by a factor of four while maintaining precise synaptic balance. We then simulated responses to six input patterns with response amplitudes and correlations matching those of activity patterns evoked by the experimental panel of amino acid and bile acid odors across mitral cells of the olfactory bulb. Virtual odors were thus separated into inputs corresponding to amino acids (odors A1, A2, A3) or bile acids (B1, B2, B3) based on their correlations. In networks without assemblies, dE was higher between representations of A-odors than between representations of B-odors, and highest across odor classes (Fig. 4d). dM was also higher between A-odor than between B-odor representations (Fig. 4g). Across odor classes, however, dM was higher in the direction from vectors representing A-odors to reference distributions (manifolds) representing B-odors (${\text{dM}}_{\text{A}\to \text{B}}$) than in the opposite direction (${\text{dM}}_{\text{B}\to \text{A}}$) (Fig. 4g).

Introducing assemblies representing two A-odors increased dE between manifolds representing these “learned” and other odors (Fig. 4e,f), which can be attributed to a modest increase in firing rates of neurons within but not outside an assembly^30^. This effect generalized partially to the third A-odor, but not to B-odors. dM was increased for learned odors specifically in the direction ${\text{dM}}_{\text{A}\to \text{B}}$ (from A-odors to B-odors), indicating that manifold geometry changed non-uniformly (Fig. h,i). This asymmetric change in dM also generalized to the third A-odor, while no obvious effects were observed on distances between B-odors. In the computational model, the introduction of autoassociative memories therefore resulted in specific geometrical modifications of manifolds representing learned and related odors, as summarized in Fig. 4j.

### Learning-related modifications of representational manifolds in pDp

**Fig. 5 ∣:**
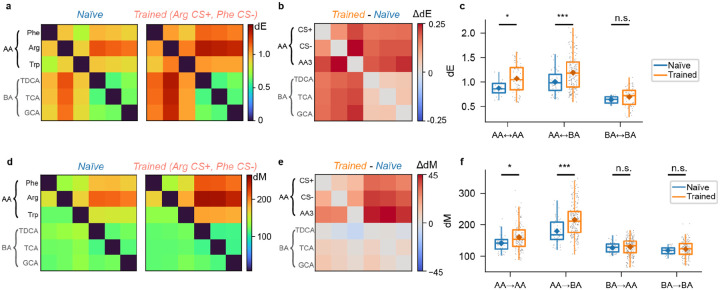
Effects of behavioral training on distances between representational manifolds pDp of juvenile zebrafish **a,** Pairwise dE between manifolds representing different amino acid (AA) and bile acid (BA) odors in naïve juvenile fish (left; averaged over 6 fish) and in fish trained to discriminate Arg (${\text{CS}}^{+}$) from Phe (${\text{CS}}^{-}$; averaged over 9 fish). **b,** Difference in dE between trained (different training groups; n = 25) and naïve fish (n = 6). AA odors were reordered by task relevance (${\text{CS}}^{+}$, ${\text{CS}}^{-}$, neutral third amino acid AA3) in matrices from trained and naive fish prior to averaging of difference matrices over training groups. **c,** dE between pairs of manifolds as a function of odor categories. dE between manifolds representing two amino acids (AA↔AA), and between manifolds representing one amino acid and one bile acid (AA↔BA), was significantly higher in trained fish (AA↔AA: 1.07 ± 0.30, mean ± SD; n = 75 pairs from N = 25 fish; AA↔BA: 1.19 ± 0.34, n = 225 pairs from N = 25 fish) than in naïve fish (AA↔AA: 0.87 ± 0.16, n = 18 pairs from N = 6 fish, Mann–Whitney U test, P = 0.011; AA↔BA: 1.00 ± 0.24, n = 54 pairs from N = 6 fish, P = 0.0002). dE between manifolds representing two bile acids (BA↔BA) was not significantly different between trained (0.70 ± 0.19, n = 75 pairs from N = 25 fish) and naïve fish (0.64 ± 0.07, n = 18 pairs from N = 6 fish, Mann–Whitney U test, P = 0.108). The sequence of relative changes in dE was AA↔AA (mean ± SD 23 ± 8%) > AA↔BA (19 ± 7%) > BA↔BA (9 ± 3%). **d, e,** pairwise distances matrices (**d**) and sorted difference matrix (**e**) for dM. **f,** Pairwise dM as a function of odor categories. Significantly higher dM was observed in trained fish between manifolds representing amino acids (AA→AA; trained: 160 ± 42, n = 150 pairs from N = 25 fish; naïve: 141 ± 27, n = 36 pairs from N = 6 fish, Mann–Whitney U test, P = 0.011) and in the direction from amino acids to bile acids (AA↔BA; trained: 216 ± 53, n = 225 pairs from N = 25 fish; naïve: 180 ± 39, n = 54 pairs from N = 6 fish, Mann–Whitney U test, P = 1.7 × 10^−6^). No significant differences in dM between trained and naïve fish were observed in the direction from bile acids to amino acids (BA→AA; trained: 129 ± 27, n = 225 pairs from N = 25 fish; naïve: 127 ± 16, n = 54 pairs from N = 6 fish, Mann–Whitney U test, P = 0.174) and between manifolds representing bile acids (BA→BA; trained: 122 ± 26, n = 150 pairs from N = 25 fish; naïve: 118 ± 12, n = 36 pairs from N = 6 fish, Mann–Whitney U test, P = 0.26). The sequence of relative change in dM was AA→BA (mean ± SD 20 ± 7%) > AA→AA (13 ± 4%) > BA→BA (3 ± 1%) > BA→AA (1.6 ± 0.4%).

To determine how discrimination training modified representational manifolds in pDp we first compared matrices of dE and dM in naïve and trained fish (Fig. 5a,d). dE was increased between the ${\text{CS}}^{+}$, the ${\text{CS}}^{-}$, and the third amino acids (“AA3”) but remained almost constant between amino acids and bile acids (Fig. 5b). This pattern was observed in all individual training groups. Matrices of dM were asymmetric. In particular, dM was higher in the direction from amino acids to bile acids (${\text{dM}}_{\text{AA}\to \text{BA}}$) than in the opposite direction (${\text{dM}}_{\text{BA}\to \text{AA}}$)(Fig. 5e). In all groups of trained fish, dM was increased non-uniformly between different classes of odor pairs (amino acids versus amino acids [AA-AA], amino acids versus bile acids [AA-BA], bile acids versus bile acids [BA-BA]). Among amino acids (AA-AA), dM was significantly increased between all odors (${\text{CS}}^{+}$, ${\text{CS}}^{-}$ and AA3) while no significant increase was observed between representations of bile acids (BA-BA; Fig. 5f). Largest increases were found between representations of amino acids and bile acids (AA-BA). These increases occurred selectively in the direction from amino acid activity vectors to bile acid reference distributions (${\text{dM}}_{\text{AA}\to \text{BA}}$), whereas dM in the opposite direction (${\text{dM}}_{\text{BA}\to \text{AA}}$) remained almost constant. Hence, effects of discrimination training on representational manifolds were consistent with model predictions.

Together with previous computational analyses^30^, these observations indicate that training had two major effects on representational manifolds of conditioned and related odors: first, manifold centers became more distant from each other and from other manifolds, as revealed by the increase in dE. Second, manifold geometry changed non-uniformly. The finding that training increased ${\text{dE}}_{\text{AA-BA}}$ but not ${\text{dM}}_{\text{BA}\to \text{AA}}$ implies that representational manifolds for amino acids expanded in the direction from amino acids to bile acids because the increase in dE must be accompanied by an increase in variability along the same direction. However, the increase in variability did not exceed the increase in dE and, therefore, did not compromise discriminability. In other directions, however, dM increased, indicating that variability increased less than dE, implying a non-uniform change in manifold geometry. As dM directly reflects the linear separability of a given pattern from a reference distribution, the asymmetric and A-selective increase enhances the linear discriminability of activity vectors representing learned or related odors from manifolds representing other odors.

### Changes in manifold geometry support pattern classification

**Fig. 6 ∣:**
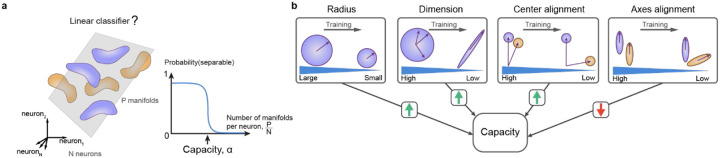
Manifold capacity, effective geometric measures, and summary of observations **a**, Manifold capacity: schematic illustration. Left: P manifolds, whose geometries satisfy a given distribution, are embedded in an N-dimensional state space (representing the joint activity of N neurons) and randomly assigned binary labels (1 or −1). The manifold capacity α is the critical number of manifolds per dimension (P/N) above which the manifolds are no longer linearly separable according to their labels (right). **b**, Geometric measures affecting manifold capacity: schematic illustration. Radius measures how far the manifold extends from its center. A decrease in radius indicates a more compact manifold. Dimension measures the number of directions in which the manifold extends. A decrease in dimension indicates a “flatter” manifold. Alignment measures are defined for pairs of manifolds. Center alignment measures the correlation between manifold centers. Axes alignment measures the amount of directional similarity between manifolds. A decrease in axes alignment indicates that manifolds extend in more different directions. Note that these geometric measures are effective measures computed after reweighting manifold points based on their importance in classification, and thus can be different from the intrinsic geometric measures of the manifolds alone. Green and red arrows specify the relationships between each geometric measure and manifold capacity. Decreases in radius, dimension and center alignment all lead to an increase in the capacity (green arrows). Decrease in axes alignment leads to a decrease in capacity (red arrow). Changes shown in the schematic summarize the observed effects of discrimination training on geometrical parameters and manifold capacity in pDp.

The increase in dM in the direction from learned to other odors suggests that learning-related changes in manifold geometry support pattern classification. To further investigate this hypothesis we used the analytical framework of GCMC^36^. This framework quantifies the separability of manifolds using manifold capacity^9,14,35^, a metric that assesses how efficiently manifolds are stored in neural state space for retrieval via linear readout (Fig. 6a). Specifically, higher manifold capacity indicates greater discriminability of manifolds. In other words, a downstream neuron, modeled as a linear sum followed by thresholding, can distinguish between manifolds by accessing a smaller number of upstream neurons. See ^36^ for additional interpretations of manifold capacity.

GCMC uses a set of parameters to describe geometrical features and correlations of manifolds (Fig. 6b). To compute manifold capacity, manifolds are repeatedly sampled from a distribution defined by these parameters and embedded in a state space of a given dimensionality. Individual manifolds are randomly assigned a binary label (1 or −1) and linear classifiers are trained to separate sets of manifolds with different labels. Manifold capacity is then defined as the maximum number of manifolds per dimension that can be embedded until linear classification fails (Fig. 6a). As GCMC theory analytically links geometrical parameters to classification error it provides a mathematical framework to understand how manifold capacity depends on geometrical features of representational manifolds and their correlations.

**Fig. 7 ∣:**
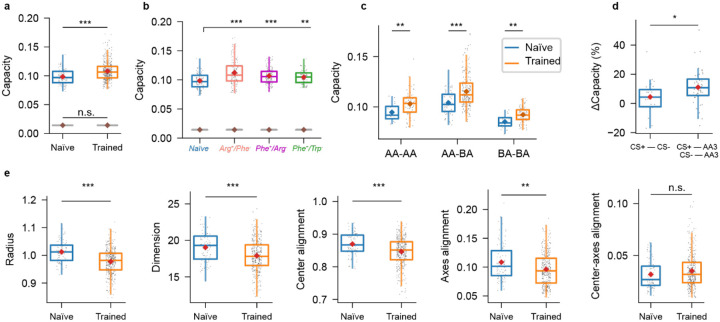
Effects of odor discrimination training on manifold capacity and geometry in juvenile zebrafish **a**, Manifold capacity in naïve and trained groups. Gray: manifolds after shuffling of labels. Naïve (mean ± SD): 0.098 ± 0.013, n = 90 odor pairs from N = 6 fish; trained: 0.108 ± 0.017, n = 225 odor pairs from N = 25 fish (Mann–Whitney U test, P = 2.9 × 10^−7^). Shuffled naïve: 0.014 ± 0.0001; shuffled trained: 0.014 ± 0.0001 (Mann–Whitney U test, P = 0.333). **b**, Manifold capacity in naïve fish and three groups of fish trained on different odor-reward associations. Manifold capacity was significantly increased in all training groups. Arg^+^/Phe^−^ (mean ± SD): 0.112 ± 0.021, n = 135 odor pairs from N = 9 fish; Phe^+^/Arg^−^: 0.107 ± 0.013, n = 150 odor pairs from N = 10 fish; Phe+/Trp: 0.104 ± 0.011, n = 90 odor pairs from N = 6 fish. Differences in manifold capacity were statistically significant between the naïve group (0.098 ± 0.013, n = 90 odor pairs from N = 6 fish) and each of the three training groups (Kruskal–Wallis test, n = 465, d.f. = 3, H = 31.48, P = 6.7 × 10^−7^; post-hoc Dunn test: Arg^+^/Phe^−^, P = 7.0 × 10^−16^; Phe+/Arg−, P = 1.1 × 10^−10^; Phe+/Trp, P = 1.0 × 10^−5^). Gray: manifold capacity after shuffling manifold labels was not significantly different between the naïve and trained groups (Kruskal–Wallis test, P = 0.302). **c**, Manifold capacity was significantly increased for different classes of odor pairs. Amino acids versus amino acids (AA-AA; mean ± SD), naïve: 0.095 ± 0.009, n = 18 pairs from N = 6 fish; trained: 0.103 ± 0.011, n = 75 pairs from N = 25 fish, Mann–Whitney U test, P = 0.003. Amino acids versus bile acids (AA-BA), naïve: 0.104 ± 0.012, n = 54 pairs from N = 6 fish; trained: 0.115 ± 0.016, n = 225 pairs from N = 25 fish, Mann–Whitney U test, P = 3.4 × 10^−6^. Bile acids versus bile acids (BA-BA), naïve: 0.085 ± 0.007, n = 18 pairs from N = 6 fish; trained: 0.092 ± 0.008, n = 75 pairs from N = 25 fish, Mann–Whitney U test, P = 0.001. **d,** Relative increase in manifold capacity was significantly higher between each of the conditioned odors (${\text{CS}}^{+}$, ${\text{CS}}^{-}$) and the third amino acid (AA3; 11.1 ± 11.7 % 50 pairs from N = 25 fish) than between the conditioned odors (mean ± SD 4.4 ± 10.8 %, 25 pairs from N = 25 fish; Mann–Whitney U test, P = 0.011). **e,** Geometric measures in the naïve and trained groups. Radius in the trained group (mean ± SD 0.978 ± 0.045, n = 225 pairs from N = 25 fish) was significantly lower than in the naïve group (1.012 ± 0.038, n = 90 pairs from N = 6 fish; Mann–Whitney U test, P = 1.3 × 10^−10^). Dimension in the trained group (17.9 ± 2.1) was significantly lower than in the naïve group (19.0 ± 2.0; Mann–Whitney U test, P = 1.0 × 10^−5^). Center alignment in the trained group (0.847 ± 0.043) was significantly lower than in the naïve group (0.870 ± 0.033; Mann–Whitney U test, P = 5.2 × 10^−6^). Axes alignment in the trained group (0.096 ± 0.029) was significantly lower than in the naïve group (0.108 ± 0.031; Mann–Whitney U test, P = 0.001). Center-axes alignment in the trained group (0.035 ± 0.017) was not significantly different from the naïve group (0.032 ± 0.013; Mann–Whitney U test, P = 0.095).

GCMC theory expresses manifold capacity as a function of five parameters that measure interpretable geometrical features of manifolds and their correlations (Fig. 6b): (1) effective manifold radius (related to “compactness”), (2) dimension (related to “flatness”), (3) center alignment (related to “pattern correlation”, i.e., the correlation between activity vectors representing manifold centers), (4) axes alignment (related to the “relative orientation” of manifolds), and (5) center-axes alignment (related to the interaction of “pattern correlation and relative orientation”). These measures provide a statistical description of representational manifolds that is linked to manifold capacity through a mathematical characterization of how linear readout interacts with manifold structure. The relationship between manifold capacity and parameters (1) – (4) is monotonic: manifold capacity increases when effective radius decreases, effective dimension decreases, effective center alignment decreases and effective axes alignment increases (Fig. 7c). The effect of center-axes alignment on manifold capacity (5) is more complex and depends on the other parameters.

We quantified manifold capacity based on sets of activity vectors across all pDp neurons within a 7 s time window starting 1 s after the odor response onset, pooled over the three trials for each odor. Across all odors and training groups, manifold capacity was significantly higher in trained (0.108 ± 0.017; n = 375 odor pairs from N = 25 fish; mean ± SD) than in naïve fish (0.098 ± 0.013; P = 2.9 × 10^−7^; n = 90 odor pairs from N = 6 fish; Fig. 7a). Shuffling manifold labels across all activity patterns substantially decreased the resulting capacity and abolished differences between naïve and trained groups (Naïve: 0.014 ± 0.0001; Trained: 0.014 ± 0.0001; P = 0.33). Manifold capacity was increased in each individual training group in comparison to naïve fish (Fig. 7b), indicating that training consistently enhanced the separability of representational manifolds.

To examine whether changes in manifold capacity were specific to an odor category we separately analyzed three classes of odor pairs: AA-AA (amino acids versus amino acids), AA-BA (amino acids versus bile acids), and BA-BA. For all three classes, manifold capacity was significantly higher in trained than in naïve fish (AA-AA: P = 0.003; AA-BA P = 3.4 × 10^−6^; BA-BA P = 0.001; Fig. 7c). Largest relative differences in manifold capacity were observed for AA-BA pairs (10.80 ± 15.19 %), followed by AA-AA (8.84 ± 12.22 %) and BA-BA pairs (8.20 ± 8.83 %).

We next tested the hypothesis that training primarily modified manifolds representing the ${\text{CS}}^{+}$ or ${\text{CS}}^{-}$. We first compared pairs of manifolds representing conditioned odors to pairs of manifolds representing one learned amino acid (${\text{CS}}^{+}$ or ${\text{CS}}^{-}$) and the third amino acid in the odor set (AA3). The relative increase in manifold capacity was significantly higher for classification of a conditioned odor versus AA3 (11.1 ± 11.7 %; P = 0.011; Fig. 7d, Supplementary Fig. S2) than for classification of the ${\text{CS}}^{+}$ versus the ${\text{CS}}^{-}$ (4.4 ± 10.8 %). These observations indicate that training enhanced the separability of both conditioned odors from other odors, as well as from each other. We therefore conclude that specific geometrical modifications of representational manifolds support pattern classification, particularly the discrimination of conditioned from other stimuli.

To further understand the underlying modifications of representational manifolds we analyzed the contributions of different geometrical parameters (Fig. 6b, 7e). In trained fish, the effective radius, dimensionality, center alignment and axes alignment were all significantly lower than in naïve fish (radius: 1.01 ± 0.04 [naïve] versus 0.98 ± 0.04 [trained]; P = 1.3 × 10^−10^; dimensionality: 19.0 ± 2.0 versus 17.9 ± 2.1; P = 1.0 × 10^−5^; center alignment: 0.87 ± 0.03 versus 0.85 ± 0.04; P = 5.2 × 10^−6^; axes alignment: 0.108 ± 0.031 versus 0.096 ± 0.028; P = 0.001), while center-axes alignment was not significantly different (0.032 ± 0.013 versus 0.035 ± 0.017; P = 0.095). The increased manifold capacity was therefore due to decreases in effective manifold radius, dimensionality, and center alignment that outweighed opposite effects of changes in axes alignment on manifold capacity. A decrease in effective radius indicates that manifolds become more “compact”, thus enhancing their separability, while a decrease in effective dimensionality implies that manifolds become more constrained in specific state space dimensions. A decrease in center alignment supports pattern classification by decorrelating representations. Our results indicate that these are the most prominent changes that improve the capacity of manifolds for pattern classification after training (Fig. 6b).

The effective radius and dimensionality of manifolds were decreased for all classes of odor pairs (Supplementary Fig. S2; AA-AA radius: 1.02 ± 0.03 [naïve] versus 0.99 ± 0.04 [trained], P = 0.0004; AA-AA dimensionality: 19.5 ± 1.5 versus 18.4 ± 1.7, P = 0.018; AA-BA radius: 1.00 ± 0.03 versus 0.96 ± 0.04, P = 4.2 × 10^−8^; AA-BA dimensionality: 18.2 ± 1.7 versus 16.9 ± 1.8, P = 5.4 × 10^−5^; BA-BA radius: 1.05 ± 0.03 versus 1.02 ± 0.03, P = 0.0002; BA-BA dimensionality: 21.2 ± 1.3 versus 20.2 ± 1.5, P = 0.013), indicating that training reduced within-manifold variability relevant for classification in all odor categories. Center alignment was decreased significantly for AA-BA (0.86 ± 0.03 versus 0.83 ± 0.04, P = 3.2 × 10^−5^) and BA-BA (0.89 ± 0.03 versus 0.87 ± 0.03, P = 0.004) pairs, but not for AA-AA pairs (0.88 ± 0.03 versus 0.87 ± 0.03, P = 0.23). Axes alignment was decreased only for AA-BA (0.099 ± 0.026 vs 0.087 ± 0.024, P = 0.003), but not for AA-AA (0.12 ± 0.03 vs 0.11 ± 0.02, P = 0.30) and BA-BA (0.12 ± 0.04 vs 0.11 ± 0.03, P = 0.10). Hence, training had somewhat different effects on manifolds representing conditioned inputs, related inputs, and dissimilar inputs, but decreases in effective manifold radius (“compactness”) and dimensionality (“flatness”) occurred consistently.

### Experience-dependent modification of representational manifolds in adult fish

**Fig. 8 ∣:**
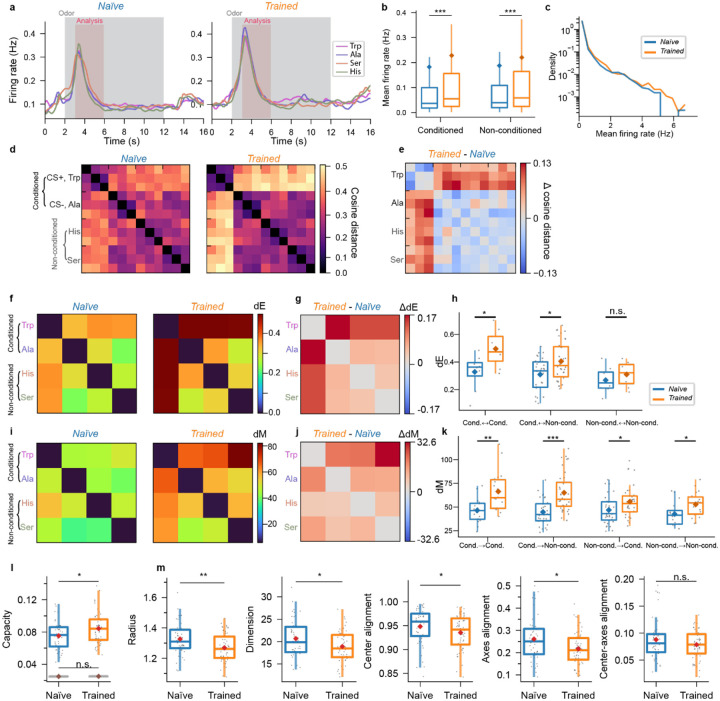
Effects of discrimination training on odor representations in pDp of adult zebrafish **a,** Mean time course of inferred firing rates in response to each odor, averaged across all neurons and trials and fish, in naïve (N = 8 fish; left) and trained adult zebrafish (N = 9; right). Gray shading indicates odor presentation (10 s); red shading shows time window for standard analyses (3 s). **b,** Response amplitude evoked by conditioned (${\text{CS}}^{+}$: Trp, ${\text{CS}}^{-}$: Ala; left) and non-conditioned odors (Ser, His; right) was significantly higher in trained than in naïve fish (Conditioned odors [mean ± SD], trained: 0.23 ± 0.55, n_odor_ = 2, n = 6092 neurons from N = 9 fish; naïve: 0.18 ± 0.49, n_odor_ = 2, n = 5164 neurons from N = 8 fish; Mann–Whitney U test, P = 7.6 × 10^−88^; non-conditioned odors, trained: 0.22 ± 0.52, n_odor_ = 2, n = 6092 neurons from N = 9 fish; naïve: 0.19 ± 0.49, n_odor_ = 2, n = 5164 neurons from N = 8 fish; Mann–Whitney U test, P = 4.1 × 10^−66^). c, Histogram of response amplitudes for conditioned odors in naïve (blue) and trained (orange) fish. **d,** Cosine distance between activity patterns evoked by different odors in individual trials, averaged over fish (left: naïve; right: trained fish). **e,** Difference between the cosine distance matrices in (**d**). **f,** Matrix of dE averaged over naïve (left) and trained fish (right). **g,** Difference between dE matrices (**f**). **h,** dE between pairs of manifolds representing odors from different categories. dE between ${\text{CS}}^{+}$ and ${\text{CS}}^{-}$ (Cond.↔Cond.) was significantly higher in trained (mean ± SD: 0.49 ± 0.13, n = 9 pairs from N = 9 fish) than in naïve fish (0.33 ± 0.13, n = 8 pairs from N = 8 fish, Mann–Whitney U test, P = 0.046). dE between conditioned and non-conditioned (Cond.↔Non-cond.) was also significantly higher in trained (0.41 ± 0.14, n = 36 pairs from N = 9 fish) than in naïve fish (0.31 ± 0.11, n = 32 pairs from N = 8 fish, Mann–Whitney U test, P = 0.014). dE between non-conditioned odors (Non-cond.↔Non-cond.) was not significantly different (trained: 0.31 ± 0.09, n = 9 pairs from N = 9 fish; naïve: 0.27 ± 0.09, n = 8 pairs from N = 8 fish, Mann–Whitney U test, P = 0.423). The sequence of relative changes in dE was Cond.↔Cond. (mean ± SD 48 ± 23%) > Cond.↔Non-cond. (32 ± 16%) > Non-cond.↔Non-cond. (15 ± 7%). **i,** Matrix of dM averaged over naïve (left) and trained fish (right). **j,** Difference between dM matrices (i). **k,** dM between manifolds representing odors from different categories. dM between conditioned odors (Cond.→Cond.) was higher in trained (66 ± 21, n = 18 pairs, from N = 9 fish) than in naïve (46 ± 13, n = 16 pairs, from N = 8 fish, Mann–Whitney U test, P = 0.004). dM from conditioned to non-conditioned odors (Cond.→Non-cond.) was higher in trained (65 ± 20, n = 36 pairs from N = 9 fish) than in naïve (45 ± 14, n = 32 pairs from N = 8 fish, Mann–Whitney U test, P = 1.7 × 10^−5^). dM from non-conditioned to conditioned odors (Non-cond.→Cond.) was higher in trained (56 ± 17, n = 36 pairs from N = 9 fish) than in naïve (41 ± 13, n = 32 pairs from N = 8 fish, Mann–Whitney U test, P = 0.027). dM from non- conditioned to non-conditioned odors (Non-cond.→Non-cond.) was higher in trained (mean ± SD 53 ± 13, n = 18 pairs from N = 9 fish) than in naïve fish (43 ± 13, n = 16 pairs from N = 8 fish, Mann–Whitney U test, P = 0.028). The sequence of relative changes in dM was Cond.→Non-cond. (mean ± SD 44 ± 63%) > Cond.→Cond. (43 ± 61%) > Non-cond.→Cond. (37 ± 60%) > Non-cond.→Non-cond. (23 ± 48%). **l,** Manifold capacity (gray: manifolds with labels shuffled). Capacity in trained fish (mean ± SD 0.084 ± 0.019, n = 54 pairs from N = 9 fish) was higher than in naïve fish (0.075 ± 0.017, n = 48 pairs from N = 8 fish; Mann–Whitney U test, P = 0.022). Differences after shuffling of labels were not significant (trained: 0.025 ± 0.0003; naïve: 0.025 ± 0.0003; Mann–Whitney U test, P = 0.066). **m,** Geometric measures of manifolds in naïve and trained fish. Radius was significantly lower in trained (1.27 ± 0.09, n = 54 pairs from N = 9 fish) than in naïve fish (1.33 ± 0.11, n = 48 pairs from N = 8 fish; Mann–Whitney U test, P = 0.007). Dimension was significantly lower in trained (18.9 ± 3.4) than in naïve fish (20.7 ± 4.1; Mann– Whitney U test, P = 0.040). Center alignment was lower in trained (0.935 ± 0.036) than in naïve fish (0.948 ± 0.038; Mann–Whitney U test, P = 0.045). Axes alignment was significantly lower in trained (0.217 ± 0.075) than in naïve fish (0.261 ± 0.098; Mann–Whitney U test, P = 0.026). Center-axes alignment in trained fish (0.079 ± 0.026) was not significantly different from the naïve group (0.088 ± 0.037; Mann–Whitney U test, P = 0.571).

We next examined odor representations in adult zebrafish, where pDp is substantially larger. Effects of training may thus be expected to generalize less from conditioned stimuli to related stimuli because a larger network allows for more specific local modifications of manifold geometry. Adult zebrafish were trained in our discrimination task using Trp as ${\text{CS}}^{+}$ and alanine (Ala) as ${\text{CS}}^{-}$. After training, activity patterns were measured in response Trp, Ala, and two additional amino acids, serine (Ser) and histidine (His), that are structurally similar to the conditioned odors and evoke correlated responses in the adult olfactory bulb^48,56^. As observed in juvenile fish, training slightly increased odor responses and strong responses were observed more frequently (Fig. 8a-c). In naïve fish, the similarity between activity patterns was generally low (high cosine distance), even between responses to the same odors in different trials (Fig. 8d, left). In trained fish, the similarity of activity patterns was higher for repeated applications of the same odors but slightly lower for dissimilar stimuli, resulting in a more pronounced block structure in the distance matrix (Fig. 8d,e). Hence, odor representations in pDp were somewhat more distinct in trained fish but response variability remained high in comparison to odor responses in the olfactory bulb^39,48-50^. Basic effects of training on odor-evoked activity in adult pDp were therefore similar to those in juvenile fish, possibly with more odor-specific changes in pattern similarity.

As observed in juvenile fish, training significantly increased dE (Fig. 8f-h) and dM (Fig. i-k) in adult pDp. The increase in dE was largest between the conditioned odors (Trp, Ala) while the increase in dE between non-conditioned odors was smallest and not statistically significant (Fig. 8h). dM was increased predominantly in the direction from conditioned to other odors (i.e., from an activity vector representing a conditioned odor to a distribution of activity vectors representing another conditioned or a novel odor; Fig. 8k), with largest increases in the direction from the ${\text{CS}}^{+}$ to non-conditioned odors (${\text{dM}}_{\text{Trp}\to \text{Ser}}$, ${\text{dM}}_{\text{Trp}\to \text{His}}$). Increases in dM in the opposite direction or between non-conditioned odors were smaller. Hence, training resulted in an asymmetric increase in dM that primarily enhanced the discriminability of responses to conditioned odors. Training therefore selectively modified manifolds representing the ${\text{CS}}^{+}$ and ${\text{CS}}^{-}$ even relative to representations of related amino acid.

Manifold capacity in adult pDp was significantly higher in the trained fish as compared to the naïve (Fig. 8l). Parameter-specific analyses showed that the effective radius, dimensionality, center alignment and axes alignment were all significantly lower in trained as compared to naïve fish (Fig. 8m). Hence, as observed in juveniles, the increase in manifold capacity can be attributed to changes in the effective radius, dimensionality and center alignment of representational manifolds, which collectively outweighed a negative contribution of changes in axes alignment. Together, these observations indicate that training resulted in consistent geometrical modifications of representational manifolds in juvenile and adult zebrafish that enhance the capacity for pattern classification.

### Manifold geometry predicts discrimination behavior

**Fig. 9 ∣:**
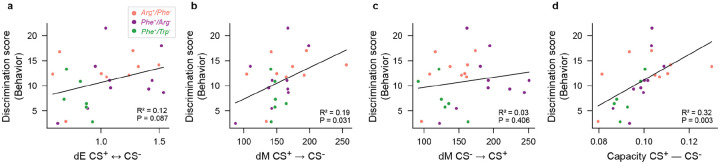
Separation of representational manifolds predicts odor discrimination behavior **a**, Discrimination score quantifying behavioral discrimination between ${\text{CS}}^{+}$ and ${\text{CS}}^{-}$ as a function of dE between manifolds representing the ${\text{CS}}^{+}$ and ${\text{CS}}^{-}$ (${\text{dE}}_{\text{CS}+,\text{CS}-}$). Each datapoint represents one fish; colors correspond to different training groups (Arg^+^/Phe^−^, Phe^+^/Arg^−^, Phe^+^/Trp; legend). The correlation was positive but not significant (R^2^ = 0.12, P = 0.087). **b**,**c**, Significant correlation between dM from ${\text{CS}}^{+}$ to ${\text{CS}}^{-}$ (${\text{dE}}_{\text{CS}+\to \text{CS}-}$) and behavioral odor discrimination (R^2^ = 0.19, P = 0.031; **b**), but not between behavioral odor discrimination and ${\text{dE}}_{\text{CS}-\to \text{CS}+}$ (R^2^ = 0.03, P = 0.406; **c**). **d**, Signsificant positive correlation between manifold capacity, computed based on neural manifolds representing ${\text{CS}}^{+}$ and ${\text{CS}}^{-}$, and behavioral odor discrimination (R^2^ = 0.32, P = 0.003).

To examine whether the geometry of representational manifolds in pDp has a direct influence on behavior we asked whether geometrical features of manifolds predict behavioral odor discrimination. The Euclidean distance between manifolds representing conditioned odors (${\text{dE}}_{\text{CS}+,\text{CS}-}$) was positively correlated with the behavioral discrimination score ζ (difference between appetitive behavioral response to ${\text{CS}}^{+}$ versus ${\text{CS}}^{-}$) across individual juvenile fish from all training groups but this correlation was not statistically significant (P = 0.087; Fig. 9a). A significant positive correlation was found between ${\text{dE}}_{\text{CS}+\to \text{CS}-}$ (Mahalanobis distance between an activity vector representing ${\text{CS}}^{+}$ from the manifold representing ${\text{CS}}^{-}$) and ζ (R^2^ = 0.19; P = 0.031; Fig. 9b), while the correlation between ${\text{dE}}_{\text{CS}-\to \text{CS}+}$ and ζ was not statistically significant (P = 0.406; Fig. 9c). The highest significant correlation was detected between manifold capacity, calculated based on representations of the ${\text{CS}}^{+}$ and ${\text{CS}}^{-}$, and ζ (R^2^ = 0.32; P = 0.003; Fig. 9d). Together, these results provide strong evidence for a direct relationship between geometrical features of representational manifolds relevant for pattern classification and learned olfactory discrimination behavior.

## DISCUSSION

### Neural manifolds in an autoassociative network

To explore the organization of information in an autoassociative memory network we examined effects of olfactory discrimination training on neural manifolds in pDp. Activity evoked by novel or conditioned odors did not exhibit obvious signs of classical or chaotic attractor states. Hence, while our experimental observations do not strictly rule out the existence of attractor states in pDp, they are inconsistent with the classical view of primary olfactory cortex as an autoassociative memory network that classifies odors by convergent dynamics.

In principle, associative learning may be mediated by any mechanism that maps relevant inputs to defined activity subspaces according to their semantic relations. For example, activity patterns representing task-relevant sensory stimuli (inputs) may be associated with different task outcomes (semantic categories) by mapping them to separate neural manifolds (activity subspaces). Stimuli may thus be classified by a systematic organization of neural manifolds according to semantic categories, even if manifolds are not attractors. In fact, the formation of manifolds representing semantic categories is the basis for pattern classification by convolutional networks, which lack attractor dynamics by design^13,57^. Unlike such convolutional networks, however, biological memory networks are often recurrent, pre-structured, and modified by local learning rules. The small size of pDp together with computational modeling and GCMC theory allowed us to directly analyze representational manifolds and their plasticity in an autoassociative area of the vertebrate brain.

The notion that neural manifolds organize relevant information in neuronal state space implies that geometrical and statistical properties of manifolds are directly relevant for neural computation^9^. Quantitative analyses of features such as the effective radius, dimension and alignments of neural manifolds should thus drive insights into neuronal computations at the population level, similar to analyses of receptive fields at the single-neuron level. Consistent with this notion, defined manifold features were modified systematically by discrimination training, demonstrating that representational manifolds are interpretable objects that capture computationally relevant structure in neuronal population activity. The finding that manifold capacity predicted discrimination behavior across individuals further indicates that manifold geometry is directly linked to behaviorally relevant neuronal computations. These observations imply that GCMC theory provides valuable tools to extract computationally relevant information about neural manifolds from large-scale measurements of population dynamics.

### Representational manifolds and olfactory memory

Our results do not support the classical view of pDp as an autoassociative memory network that classifies sensory inputs by convergent population dynamics. Rather, our findings indicate that pDp contains a continuous representation of odor space that is modified, but not discretized, by constraining activity onto neural manifolds as a function of experience. This scenario is consistent with multiple experimental observations in pDp and piriform cortex: (1) Training of animals in olfactory memory tasks led to the emergence of task-specific responses but the continuous representation of odor space observed in naïve animals was not reorganized fundamentally (Fig. 3g,4d)^24,26,28,29^. This observation is consistent with the notion that pDp or piriform cortex do not store discrete items of information but jointly represent odor space and semantic information in continuous maps^18,19,27^. (2) Experimental results in pDp (Fig. 3g,4d) and piriform cortex revealed only minor effects of training on the variability of single-neuron responses or global population activity^24,29^. These observations are consistent with the notion that representational manifolds may constrain neuronal activity only in a subset of state space dimensions. (3) Accurate odor discrimination requires reliable readout of information from population dynamics despite high variability of neuronal activity in pDp and piriform cortex. The notion of representational manifolds can reconcile these issues because activity is constrained to subspaces and, thus, information can be retrieved reliably by integrating activity over the manifold^30,58,59^. (4) In pDp^40^, and possibly also in piriform cortex^60^, network activity enters a state of precise synaptic balance during an odor response that may not support discrete attractor states. Representational manifolds can directly account for this observation and allow for pattern classification without attractor dynamics.

Discrimination training primarily enhanced the ability to classify activity patterns representing conditioned or related odors. Modifications of representational manifolds therefore contain task-relevant information that is combined with a map of odor space, resulting in joint representations of sensory and semantic information. Previously, computational models showed that semantic information can be stored in manifold geometry by the formation of neuronal assemblies in synaptically balanced autoassociative networks^30^. Predictions of these models were fully consistent with odor-specific effects of discrimination training on dE and dM in different groups of fish. However, detailed reconstructions of network connectivity will eventually be required to understand biological mechanisms that define manifold geometry.

Behavioral training consistently increased manifold capacity and, thus, optimized representational manifolds for odor classification. Increased manifold capacity could, in part, be attributed to higher “compactness” (decreased effective radius) and lower correlation (decreased center alignment) of representational manifolds, in agreement with increases in dE and dM. Manifold capacity was further enhanced by a decrease in dimensionality. In computational models, a similar decrease in dimensionality could be attributed, in part, to a modest amplification of activity in an odor-specific direction^30^. Such a directional amplification of activity may also contribute to the decrease in axes alignment observed in pDp, which had a negative effect on manifold capacity. Hence, training may modify multiple geometrical features of manifolds with opposing effects on manifold capacity. In combination, however, positive effects on manifold geometry outweighed negative contributions, resulting in an overall enhancement of the capacity for task-relevant odor classification.

Importantly, geometrical features of manifolds, particularly manifold capacity, predicted behavioral odor discrimination. Hence, geometrical features critical for pattern classification were directly related to behavioral odor discrimination, consistent with the hypothesis that semantic information accumulated in the geometry of representational manifolds is read out by neural classifiers controlling behavior.

### Putative computational functions of representational manifolds

Neural manifolds can support the classification of distinct pattern categories even when the representation of sensory and semantic information is continuous. In computational models, for example, information about learned odors can be retrieved efficiently by integrating activity over assembly neurons^30,59^. In our analysis of dE and dM, we found that training enhanced the ability to classify conditioned odors even when activity was read out from subsets of neurons that were selected solely based on activity, indicating that information relevant for pattern classification can be retrieved by simple, biologically plausible mechanisms. The observed increase in manifold capacity further implies that fewer neurons are required for efficient classification of conditioned odors after training because the amount of relevant information per neuron increases^14,36,59^. One computational function of experience-dependent changes in manifold geometry may therefore be to facilitate the classification of meaningful sensory inputs. In addition, modifications of continuous representational manifolds support other computations such as the evaluation of metric relationships between relevant patterns^30^.

Interpretable changes in manifold capacity have recently been described also in brain areas of other species^36^. For example, manifold capacity was found to increase along the ventral stream of the visual system in datasets from monkeys and humans. In convolutional networks trained on image classification, manifold capacity increased systematically along layers prior to the final classification layer^13^. Together, these studies and our results support the assumption that neural manifolds are informative objects critical for neuronal computation, implying that the systematic analysis of manifold geometry can uncover principles of information processing in the brain.

Since pDp does not exhibit persistent activity it is unlikely to function as an integrator network, despite the anatomical similarity between piriform cortex and hippocampal area CA3. Moreover, consistent with recent observations in piriform cortex^24^, pDp does not appear to classify odor objects by convergent attractor dynamics even after learning, indicating that it is functionally more closely related to the intermediate layers than to the final classification layer of deep convolutional networks. We therefore propose that pDp establishes “semantic maps” of odor space to support behaviorally relevant sensory pattern classification and potentially other computations as part of a larger network. This view is consistent with the concept of olfactory cortex as a memory-related structure that comprises multiple recurrent brain areas^18,19^. More generally, representational learning by experience-dependent reorganization of neural manifolds may be critical for cognitive processes that are mediated by distributed computations across the pallium.

## METHODS

### Animals and transgenic lines.

Zebrafish (*Danio rerio*) were raised and kept as groups in a standard facility at 26.5–27.5 °C on a 14/10 h light/dark cycle. Juvenile fish were kept in 1.1 L tanks (Tecniplast ZB11TK). Olfactory conditioning of juvenile fish in their home tank started 28-38 days post fertilization (dpf). Juvenile fish used for calcium imaging experiments were 46 – 56 dpf with a body length of 9.0 – 13.5 mm and not selected for gender, which is not yet determined at this stage. Adult fish were 5-6 months old and not selected for gender. The naïve group consisted of 4 females and 4 males while the trained group consisted of 5 females and 4 males. Juvenile fish expressed the calcium indicator GCaMP6s pan-neuronally under the control of the alpha-tubulin promoter (Tg[alphaTubulin:GCaMP6s]^47^). Adult fish were wildtype except for Tg[dlx4/6:ChR2-YFP]^51,52^ used in optogenetic experiments. All experimental protocols were approved by the Veterinary Department of the Kanton Basel-Stadt (Switzerland).

### Ex-vivo preparation of the zebrafish brain.

#### Ex vivo preparation of the juvenile zebrafish brain.

In juvenile zebrafish, one of the olfactory bulbs and the ipsilateral side of the telencephalon were exposed to provide optical access. Before dissection, juvenile zebrafish were transferred into a beaker containing c.a. 10 mL system water at room temperature. The beaker together with the fish was cooled with ice to 4°C to anesthetize the fish until immobile. Heart rate was visually monitored and anesthesia was ensured by pinching the tail fin. The fish was the transferred into ice-cold teleost artificial cerebrospinal fluid (ACSF: 124 mM NaCl, 2 mM KCl, 1.25 mM KH_2_PO_4_, 1.6 mM MgSO_4_, 22 mM D-(+)-glucose, 2 mM CaCl_2_ and 24 mM NaHCO_3_, pH 7.2.) bubbled with O_2_/CO_2_ (95%/5%)^61^ and fully immersed in this medium during dissection. Peripheral structures (eyes, jaws, gills) were removed and the head was detached from the body. The head was pleased on a coverslip, the exposed lateral side was oriented upwards, and the preparation was stabilized using tissue glue (3M Vetbond Tissue Adhesives, No.1469SB). Fine forceps and scissors were used to expose the olfactory bulb and telencephalon by removing soft cartilage and connective tissue without damaging the olfactory nerve. Care was taken not to obstruct the nostrils. For fish with body length above 12 mm, contralateral bones were partially removed to increase exposure of the brain to aerated ACSF. The preparation was transferred together with the coverslip to a custom flow chamber for two-photon imaging that was continuously perfused with ACSF at room-temperature.

#### Ex vivo preparation of the adult zebrafish brain.

The ex vivo preparation of adult zebrafish for the electrophysiology experiment was performed as described^40,46^. Briefly, fish were anesthetized by cooling to 4°C and decapitated in ACSF. After dissection of the jaws, the eyes and the bones covering the ventral telencephalon, the dura mater over pDp was removed with fine forceps. The ventral bones covering the olfactory bulbs were removed if necessary (electrophysiological recordings from mitral cells, optogenetic silencing). After surgery, the preparation was slowly warmed up to room temperature under constant perfusion with ACSF as described for juvenile fish.

#### Injection of a synthetic calcium indicator into adult pDp.

Fish were mounted in a custom-made holder for imaging in a horizontal orientation to ensure high reproducibility of the injection procedure as described (REF Frank et al., NN paper). Bolus loading of Oregon Green 488 BAPTA-1-AM (OGB-1; ThermoFisher Scientific) was performed as described^62^ with minor modifications. 50 μg of OGB-1-AM was dissolved in 30 μL of DMSO/Pluronic F-127 (80/20; ThermoFisher Scientific), vortexed for 1 min and sonicated for 15 min before being stored in 4 μL aliquots at −20°C. Prior to each experiment, an aliquot was diluted in 15 uL of ACSF, vortexed for 1 min, sonicated for 5 min and purified for 5 min at 5000 rcf in centrifugal filter units (PVDF membrane with 0.22 μm pore size; Millipore). A slowly tapering glass pipette was pulled from a borosilicate glass capillary (borosilicate glass capillaries, 1 mm outer diameter, wall thickness 0.21 mm, capillary length 100 mm; Hilgenberg article number 1810021) with a laser puller (P-2000; Sutter). The tip was broken under a microscope (MF-900 Microforge; Narishige) to a tip diameter of approximately 4 μm.

Pressure injections were targeted using a non-resonant two-photon scanning microscope with a 20x objective that provided both a fluorescent channel for detection of OGB-1 (PMT H7422P-40MOD, Hamamatsu) and a transmitted light channel (PSD, position-sensitive detector) as described^39^. pDp was identified based on its location in the lateral telencephalon, posterior to the prominent furrow. One primary injection was made ~210 μm dorsal from the ventral-most aspect of Dp and ~130 μm from the lateral surface of Dp in two intervals of each ca. 2 min. A second injection was performed in the same entry channel of the pipette but less deep (ca. 180 μm dorsal, 60 μm lateral) for 1-2 min. Dye injection was monitored by snapshots of multiphoton images. Pressure was adjusted to prevent fast swelling of the tissue that could be caused if the applied pressure was too high. The entire injection procedure typically took 6-10 min.

After injection, the pipette was retracted from the brain and the fish head was quickly (<2 min) transferred to another holder for imaging in a sagittal orientation using a 2-photon microscope equipped for volumetric resonance scanning. The approximately sagittal orientation allowed for improved optical access to areas of pDp close to the lateral brain surface but deeper from the ventral surface. The orientation of the brain was adjusted to minimize optical aberrations due to tissue curvature. Odor application and calcium imaging started ca. 1 h after dye injection.

### Optogenetic stimulation of the olfactory bulb.

Blue light was targeted to the ipsilateral olfactory bulb through an optical fiber (200 μm diameter, Thorlabs) using a 457 nm laser (500 mW before attenuation) as described^46^. Laser intensity was adjusted to obtain 200 μW at the fiber tip. Optical stimulation was restricted to the olfactory bulb with possible minor off-target effects onto adjacent areas.

Odors were applied for 10 s through a tube in front of the ipsilateral nostril using a peristaltic pump system as described^40^. The onset of optical stimulation (300 ms) was targeted at the plateau phase of the odor response in pDp, typically 400-500 ms after response onset. Current clamp recordings from superficial mitral cells (n = 4) showed that the latency between blue light onset and cessation of action potential firing was approximately 10–20 ms. Recovery of mitral cell activity was observed approximately 500 ms after the end of the light pulse.

### Electrophysiology.

Patch-clamp recordings of neurons in pDp were performed as described^40^ using borosilicate pipettes (pipette resistance: 4–8 MΩ) and a Multiclamp 700B amplifier (Molecular Devices). Pipettes were filled with intracellular solution containing (in mM): 132 Cs methanesulfonate, 10 Na_2_-phosphocreatine, 4 MgCl_2_, 4 Na_2_-ATP, 0.4 Na-GTP, 5 L-glutathione, 0.1 EGTA, and 10 HEPES (pH 7.2, 300 mOsm; all from Sigma). Current-clamp recordings were performed with an intracellular solution containing (in mM): 129 K-gluconate, 10 HEPES (free acid), 0.1 EGTA, 4 Na_2_-ATP, 10 Na_2_-phosphocreatine, 0.3 Na-GTP, 5 L-glutathione, and 13.1 KOH (pH 7.2, 305 mOsm; all from Sigma).

Neurons were targeted using the shadow-patching technique^63^ as described^40^, with 0.05 mM Alexa488 or Alexa594 (Invitrogen) included in the internal solution using a custom-designed video-rate multiphoton microscope^45^. When the dura mater over Dp was not completely removed, the pipette was advanced through the dura mater with transient high pressure (100 mbar) to avoid contamination of the pipette tip. Before forming a seal, neurons were approached within the tissue using low pressure (20 mbar). After break-in, series resistance and input resistance were continuously monitored. To measure the cellular time constant of Dp neurons by direct hyperpolarization, brief voltage steps (500 ms, step size ΔV = −5 mV) were applied through the patch pipette during voltage clamp experiments.

Voltage traces triggered on the onset of optical stimulation or hyperpolarizing current injections were averaged for each neuron and normalized by subtracting the pre-event current (mean of the 200 ms window before stimulation onset) and dividing by the asymptote (400-500 ms after stimulation onset). The resulting traces were fitted with an exponential function to estimate the decay time constant. An equivalent procedure was used to estimate decay time constants from normalized current traces.

### 2-photon calcium imaging.

2-photon calcium imaging was performed using a custom-built multiphoton microscope^45^ using a 20x objective (NA 1.0; Zeiss) and custom-written Scanimage software^40,64^. Laser pulses for two-photon excitation were centered around 930 nm, with a temporal pulse width of 180 fs below the objective as measured with an autocorrelator (CARPE; APE Berlin). Fluorescence was detected by a GaAsP photo-muliplier tube (PMT, H7422P-40MOD; Hamamatsu) without bandpass filtering of the emitted light to maximize fluorescence yield. The average laser power was gradually adjusted to 42-52 mW for juveniles and 23-24 mW for adults at imaging planes closest to the brain surface and 4 mW higher for deepest imaging planes using a Pockels cell (350-80LA; Conoptics) synchronized with the voice coil motor used for fast z-scanning.

Imaging was performed in 8 planes (256 x 512 pixels each) at 7.5 Hz as described^45^. Ca. 7.3 of these 8 planes were scanned during the linear trajectory of the z-scanning, while the remainder was acquired during the fast flyback of the z-scanning unit. The peak-to-peak maximum extension of the imaging volume was ca. 150 μm in juveniles and 100 μm in adults, whereas the extent of the FOV in x and y was around 250 μm and 125 μm in juveniles and 200 μm and 100 μm in adults, respectively, slightly varying with the relative z-position of the plane due to remote z-scanning^45^.

After every 1 or 2 trials, the microscope stage was repositioned in order to compensate for potential drifts. For this purpose, a small z-stack of ± 6 μm around the current location (step size, 2 μm) was acquired and the optimal shift in x, y and z was determined based on the correlation with a reference stack. Sub-resolution interpolation using a Gaussian fit of the correlation values allowed to achieve a correction accuracy well below the step size of 2 μm. For most experiments, the corrected drift was substantial over the time course of an experiment. In juveniles, the total drift ranged in 10-15 μm in pDp and 5-10 μm in the OB in z-direction, each region recorded for 80-90 min. In adults, the total drift ranged in 18-25 um in z-direction over a full experiment within 50-60 min. During each trial, average projections of each imaging plane were created and inspected for artifacts (e.g., degraded signal due to bubbles or imaging plane drifts with respect to the reference stack). Defective trials were discarded and re-acquired.

### Odor application.

Amino acids (Ala, Arg, His, Phe, Ser, Trp; Sigma) were prepared as 100x stock solutions in double-distilled water, vortexed, sonicated, stored at −20°C, and diluted to a final concentration of 10^−4^ M in ACSF immediately before the experiment. Bile acid odorants (TDCA, TCA, GCA; Sigma) were also prepared as 100x stock solutions and diluted to a final concentration of 10^−5^ M. Food extract, which was used as a positive control stimulus, was generated by heating fish food (Gemma Micro 300) in double-distilled water and filtering the product through 0.22 μm pore size filters. The product was then stored as a stock solution and diluted 500x before the experiment.

Odors were applied to the nasal epithelium through a constant stream of ACSF using a computer-controlled odor-application system based on peristaltic pumps as described^40^. In experiments using juvenile fish, a circular symmetric 9-channel plastic manifold (Darwin microfluidics Manifold 9 Ports 1/4-28 PEEK, 1/16" OD) was used to combine the flow of multiple odor channels to the main ACSF perfusion channel. A bubble trap (Diba Omnifit^®^ Bubble Traps, 21940-38) was placed in the tube delivering ACSF to the manifold. In experiments using adult fish, odors were applied using a linear manifold as described^40^. The time of response onset was determined by application of fluorescein in the absence of fish and fine-adjusted based on neural activity measurements in each experiment. Both in juvenile and adult experiments, 1-3 odor applications were performed prior to the start of data acquisition to identify the odor-responsive brain region. Odors used in these pre-trials were food odor (adult fish) or a bile acid (juvenile fish).

To measure odor-evoked activity in pDp of juvenile fish, 6 odor stimuli (Phe, Arg, Trp, TDCA, TCA, GCA), a control stimulus (ACSF), and another control with no odor delivery were applied in a pseudo-random sequence. This procedure was repeated three times with different pseudo-random sequences. Odors were applied for 5 s with an inter-trial interval of at least 2 min. The same odor application procedure with new pseudo-randomized application sequences was repeated to measure odor-evoked activity in the olfactory bulb of the same fish. In experiments on adult fish, an equivalent protocol was used to measure activity in pDp using Trp, Ala, His, Ser, Food odor, TDCA, and ACSF as stimuli. Odors were applied for 10 s.

### Odor discrimination training.

Adult zebrafish were trained in an odor discrimination task for six days as described^54^. Juvenile fish were trained using a similar procedure after pre-training in the home tank as a group (Temiz et al., manuscript in preparation). Naïve fish used as controls were obtained from the same parental stock. During the group training phase, 10-15 juvenile zebrafish (28-38 days post fertilization) were transferred into a housing tank (Tecniplast 3.5L tank, ZB30TK) that was continuously perfused with system water (26.5°C, 120–130 ml/min flow rate). During each trial, a ${\text{CS}}^{+}$ or ${\text{CS}}^{-}$ odor was delivered for 30 seconds using an Arduino-controlled peristaltic pump (Adafruit Peristaltic Liquid pump). Following ${\text{CS}}^{+}$ delivery, fish food (Tetra TetraMin Flocken) was dispensed into a feeding ring floating in a specific location after a 30-second delay, whereas no food was dispensed following presentation of the ${\text{CS}}^{-}$. Fish received 7 ${\text{CS}}^{+}$ and 14 ${\text{CS}}^{-}$ applications per day, presented in an alternating sequence with 40-minute intervals.

After 11 days of group training, fish were transferred individually to small tanks (Plastic-Haus AG, 12x6x6 cm) and allowed to acclimate for 1-3 days. Subsequently, each fish underwent individual training using the same procedure as for adult animals^54^ with minor modifications to account for differences in body size. ${\text{CS}}^{+}$ and ${\text{CS}}^{-}$ odors were the same as during the group training phase. Each odor was delivered in an alternating sequence for 9 trials with a 20-minute intertrial interval. Swimming behavior was monitored by 3D video imaging throughout the individual training phase, which lasted 2-4 days. The appetitive behavior score ζ for each odor was computed as described based on quantitative analyses of swimming speed, the z-position in the water column, the presence in the reward zone, water surface sampling, the distance to odor inflow tube and rhythmic circular swimming during the 30 seconds between odor onset and reward delivery^54^. ζ scores are combined and normalized measures similar to z-scores that quantify appetitive behavior in response to each odor. The behavior discrimination score was computed as the difference between the ζ scores for the ${\text{CS}}^{+}$ and ${\text{CS}}^{-}$, summed over trials and divided by the number of individual training days.

### Processing of image data.

Automatic region of interest (ROI) extraction was performed using StarDist (https://github.com/stardist/stardist) combined with manual correction to segment neuronal somata in the juvenile calcium imaging dataset. The model was trained with 20 RGB images (512 × 512), each color channel containing the anatomy image, ΔF/F map, and spatial correlation map as input and manually segmented neuron somata as ground-truth output. The ground-truth data was obtained from the same fish line using the same microscope as in the juvenile calcium imaging experiments. The threshold for ROI detection in StarDist was adjusted to avoid cross-neuron signal contamination. The ROIs detected by StarDist were imported into a custom MATLAB program (https://github.com/fmi-basel/neuRoi). ROIs were aligned between trials with fast Fourier transform (FFT) cross-correlation and individual neurons were manually redrawn or deleted depending on the quality of trial-by-trial alignment.

The raw fluorescence traces for each neuron were extracted by averaging the pixel intensity within the ROI and subtracting the background fluorescence. The F_0_ for each neuron was taken as the median fluorescence intensity within a 2 s time window prior to stimulus onset.

Spike probability was inferred from ΔF/F traces using the CASCADE algorithm^41^. Global_EXC_7.5Hz_smoothing200ms_causalkernel waa chosen as the spike inference model. The noise level was determined by pooling ΔF/F traces during a 2s time window from all trials and used to choose the most appropriate noise level parameter used in the inference function. The output of CASCADE is the spike probability within the 133 ms time bin corresponding to the 7.5 Hz frame rate. The downstream analysis was computed based on the spike probability values. The spike probability of the CASCADE algorithm can be converted to firing rate by multiplication with the frame rate.

Segmentation of neuronal somata in the adult calcium imaging dataset was performed manually using a custom MATLAB program (https://github.com/PTRRupprecht/Drawing-ROIs-without-GUI). Spike probability was inferred using the OGB_zf_pDp_7.5Hz_smoothing200ms model from CASCADE.

### Spiking network model of pDp.

The spiking network model of pDp was the same as in a previous study^30^ except for the number of neurons and synaptic weights, to account for the smaller size of the juvenile zebrafish brain. The model of pDp consists of 1000 excitatory (E) and 250 inhibitory (I) adaptive leaky integrate-and-fire neurons with conductance-based synapses. The neurons in pDp receive inputs from 1500 excitatory mitral cells in the OB.

#### Neuronal dynamics.

The membrane potential $V_{x}$ of a neuron x in pDp evolved according to:

$$C_{X}\frac{dV_{x}}{dt}=g_{rest\;,X}(E_{rest\;,X}-V_{x})+g_{OB,x}(E_{exc}-V_{x})+\sum_{P\in \{exc\;,in\;h\}}g_{P,x}(E_{P}-V_{x})-z_{x}\delta (X,exc),$$

where X is the excitatory or inhibitory population to which x belongs. $C_{x}$ is the membrane capacitance, $g_{\text{rest},X}$ is the leak conductance, and $E_{\text{rest},X}$ is the resting potential. The capital X in the subscripts means that the same values apply to all neurons in population X. $g_{\text{OB},x}$ is the conductance of the synapse from an olfactory bulb input to neuron x and $g_{P,x}$ is the synaptic conductance from population P to neuron x. $E_{P}$ is the reversal potential of a synapse of population P. $z_{x}$ is the adaptation current if x is an excitatory neuron^65^:

$$\tau_{a}\frac{dz}{dt}=a(V_{x}-E_{rest\;,exc})-z$$

with z set to z+b after each spike. Parameters a and b were the same as in a previous model^30^.

Parameters a and b were the same as in a previous model^30^.

The conductances $g_{P,x}$ evolved according to

$$\tau_{syn\;,P}\frac{dg_{P,x}}{dt}=-g_{P,x}+\sum_{y}\;\;w_{yx}\delta (t-t_{spike\;,y}),$$

where $\tau_{\text{syn},P}$ is the synaptic time constant, $W_{yx}$ is the synaptic weight from neuron y to neuron x, and $t_{\text{spike},y}$ is the spike time of neuron y.

#### Olfactory bulb input.

The input from the olfactory bulb to pDp was modeled by directly modulating the baseline 6 Hz firing rates of mitral cells in the olfactory bulb. Odor-evoked activities in the olfactory bulb were simulated by increasing the firing rate of 150 mitral cells and decreasing the firing rate of another 75 mitral cells. The activity pattern of mitral cells for each odor was generated to approximate the response amplitudes and pattern correlations observed in experiments.

#### Network connectivity and E/I assemblies.

Connections between two neurons x and y from populations X and Y, respectively, were drawn from a Bernoulli distribution with parameter $p_{xy}$. The strength of all existing connections was set to $w_{xy}$. While parameters $p_{xy}$ are the same as in a previous model^30^, $w_{xy}$ were fitted to account for the reduced network size. To explore a broad parameter space, 4 connectivity matrices were fitted, each with 2 instantiations drawn, with parameter values shown in Table 1. The simulation results from these 8 instantiations were used in further analysis.

E/I assemblies were introduced in pDp as described^30^ to mimic olfactory learning. The E-assembly comprised the top 100 excitatory neurons receiving the highest degree of inputs from the mitral cells activated by the associated odor. The corresponding I-assembly comprised the top 25 inhibitory neurons receiving the highest degree of inputs from the E-assembly. E/I assemblies were formed by increasing the number of connections within E-assembly and between E-assembly and I-assembly. To maintain a constant number of connections, the same number of connections was eliminated between non-assembly neurons and assembly neurons. The network with assemblies in Fig. 3 has two sets of such E/I assemblies corresponding to odor A1 and A2.

### Manifold construction and distance between manifolds.

In experimental data, neural manifolds were constructed as follows: In each fish, for each odor, the spike probability of each neuron at each time point within the 3 s analysis time window from 3 trials was pooled, resulting in a total of 72 points per manifold for each odor and fish (24 timepoints × 3 trials).

In simulated data, manifolds were constructed by computing spike rate in 100 ms time windows and pooling the activity patterns across all excitatory neurons, including the assembly and non-assembly neurons, from a 2 s time window from 4 trials for each odor.

Distance between each pair of manifolds was computed in each fish. As the computation of dM involves the inversion of the covariance matrix, to ensure the invertibility, we selected subsets of 70 neurons, repeated the sampling 50 times, and averaged the resulting dM over the 50 repeats. dE was computed using the same procedure.

dE between two manifolds X and Y was defined as:

$$dE(X,Y)=\sqrt{{\left(\mu_{X}-\mu_{Y}\right)}^{T}(\mu_{X}-\mu_{Y})},$$

where $\mu_{X}=\frac{1}{|X|}\sum_{x\in X}x$ and $\mu_{Y}=\frac{1}{|Y|}\sum_{y\in Y}y$ are the centers of the manifolds X and Y, respectively.

dM with X as sample and Y as reference is defined as:

$$dM(X,Y)=\frac{1}{|X|}$$

where ∣X∣ is the number of points in the manifold X, x is the a point in the manifold X, $\mu_{Y}$ is the center of the manifold Y, and $S_{Y}$ is the covariance matrix of the manifold Y.

### Manifold capacity analysis.

#### Data preprocessing and capacity estimation.

Manifolds were constructed from experimental data as described above. The time window used to construct has a duration of 7 s, starting from the onset of the odor response. To ensure linear separability as required by GCMC, we subsampled neurons and time points. In juvenile fish, 700 neurons and 140 activity patterns were sampled for each manifold, while in adults, 400 neurons and 80 activity patterns were sampled.

Capacity was computed separately for each pair of manifolds representing an odor pair using the GCMC framework^36^. Subsampling was repeated 50 times for each manifold pair and capacity and geometric measures were averaged over the 50 repeats. As a control, manifold points were shuffled by pooling data points from both manifolds and re-assigning labels randomly prior to the calculation of capacity. The global centering and bias parameters were both set to true during capacity computation, which translated the mean of the manifold centers to the origin and allowed for the hyperplane classifier to have an offset from the origin^36^.

#### Manifold capacity: overview.

The algorithm for computing manifold capacity is detailed in ^36^. Here we briefly describe the key steps. In the manifold capacity theory (MCT), a manifold is modeled as a convex set residing in an N-dimensional state space. It is represented by its center, its K axes stretching out from the manifold center, and the set of coordinates with respect to the manifold axes, specifying the location of the points belonging to this manifold. That is, denoting the manifold by M, a point x in the manifold can be expressed as

$$x=u_{0}+\sum_{i=1}^{K}\;s_{i}u_{i},\;\;for\;x\in M,$$

where $u_{0}$ is the manifold center, $u_{i}\in R^{N}$ for 1≤i≤k<N is the set of manifold axes, and $s\in R^{K}$ is the coordinate of x with respect to the manifold axes. We use $S\subset R^{K}$ to denote the set of all possible coordinates of points contained in the manifold.

Consider P manifolds simultaneously residing in the N-dimensional state space. Each manifold $M^{\mu }$ is indexed with μ for 1≤μ≤P. Consider a set of dichotomies Y⊆{−1,1}. As derived from MCT^36^, the manifold capacity α is:

(1) $$\alpha ={\left(\frac{1}{P}\bar{\int DT\;min_{V\in A_{y}}{\left\|V-T\right\|}_{2}^{2}}\right)}^{-1},$$

where DT is the zero-mean Gaussian measure and T is a random vector with dimension N. Finally, A is a convex set of vectors reflecting the geometry of the manifold shapes:

$$A_{y}=\{V\in R^{N}\;:min_{x^{\mu }\in M^{\mu }}y^{\mu }V^{T}x\geq 0\}.$$

Define the normal vector of the linear classifier as the solution vector. V corresponds to the inner product of the solution vector with each $u_{i}^{u}$ for i∈0,…,K. The condition in the set A requires that the hyperplane is able to separate the manifolds with a margin of at least κ.

The overline denotes the average with respect to the labels y.

#### Effective geometric measures for linear classification.

According to the MCT, capacity is affected by geometric measures including effective radius, dimension, and alignments. "Effective" emphasizes that the measures are analytically connected to the capacity value; hence, this approach can be used to analyze geometrical changes underlying changes in manifold capacity. Thus, manifolds with different intrinsic geometries can have the same effective measures due to their relative configurations.

Detailed mathematical expressions of these measures are introduced in ^36^. Here we only give an intuitive overview of how they are computed.

To understand the effective geometric measures, it is important to introduce the concept of anchor points. The capacity formula (Equation 1) is a Gaussian average (over T) of a quadratic programming problem (a convex optimization problem). According to the strong duality theory, the solution of a quadratic program is equal to the solution of its dual problem. In this case, the dual problem is a function of the points from the manifolds. Concretely, let $F(T)=min_{V\in A_{y}}{\left\|V-T\right\|}_{2}^{2}$, the duality theory gives that there exists a function g such that $F(T)=g(T,x^{1}(T),\ldots ,x^{P}(T))$ for some $x^{\mu }(T)\in M^{\mu }$ for each μ. These points $x^{1}(T),\ldots ,x^{P}(T)$ are known as anchor points. As a result, the randomness of T Induces a distribution over each manifold. These distributions are the anchor point distributions. Notice that the anchor point distributions are analytically connected to the capacity value by the following equation:

$$\alpha ={(\frac{1}{P}\int \;DT\;g(T,x^{1}(T),\ldots ,x^{P}(T))).}^{-1}$$

Finally, the effective geometric measures from GCMC are simply geometric terms extracted from the function g in the above formula. For reference, the following are the intuitive definitions of the geometric measures. We refer interested readers to the GCMC paper for a comprehensive understanding.

1. Effective radius measures how far the points spread away from the manifold center, and is normalized by the length of the manifold center. It can be seen as the amplitude of internal variability of the manifold.

2. Effective dimension measures the number of directions along which the points extend, with respect to the manifold center. It represents the degree-of-freedom of the variability.

3. Effective center-alignment measures the correlation between the centers of different manifolds. It is the cosine similarity under the anchor-point geometry.

4. Effective axes-alignment measures the correlation between the internal axes of different manifolds.

5. Effective center-axes alignment measures the correlation between internal axes of one manifold and the center of another manifold.

These geometric measures were computed simultaneously with the capacity. Each odor pair from each fish is characterized with these five measures for further statistical analysis.

### Statistical and correlation analysis.

Comparisons between naïve and trained groups were performed using a Mann–Whitney U test. For multiple comparisons between naïve and trained subgroups we used a nonparametric Kruskal–Wallis test followed by a post hoc Dunn’s test for multiple comparisons with the naïve group. Reported P values are adjusted for multiple comparisons with Bonferroni correction.

The correlation between variables was evaluated using ordinary least squares (OLS) linear regression (implemented with statsmodels package in Python), treating one variable as the independent predictor and the other as the dependent outcome. The slope, coefficient of determination (R^2^), and p-value from the regression model were reported to quantify the strength and statistical significance of the relationship.

In all statistical tests (implemented in Python with scipy.stats or scikit_posthocs package), P < 0.05 was considered statistically significant. In graphical displays, standard significance levels are indicated with asterisks (n.s.: P ≥ 0.05; * P < 0.05; ** P < 0.01; *** P < 0.001).

## Supplementary Material

Supplement 1

## Figures and Tables

**Table 1.** Weight strength parameter settings (Unit: pS)

Settings	exc→exc	inh→exc	OB→exc	exc→inh	OB→inh	inh→inh
A	95	410	128	58	68	180
B	94	400	128	58	66	150
C	80	450	128	68	68	220
D	95	520	95	51	42	190
