# Supplementary material for: Representational learning by optimization of neural manifolds in an olfactory memory network
Source: bioRxiv. 2024 Nov 18:2024.11.17.623906. Preprint. [Version 1] doi: 10.1101/2024.11.17.623906 (PMC11601331; doi:10.1101/2024.11.17.623906)
Supplement: Supplement 1 [file NIHPP2024.11.17.623906v1-supplement-1.pdf]

bioRxiv preprint doi: https://doi.org/10.1101/2024.11.17.623906; this version posted November 18, 2024. The copyright holder for this preprint (which was not certified by peer review) is the author/funder, who has granted bioRxiv a license to display the preprint in perpetuity. It is made available under aCC-BY 4.0 International license.

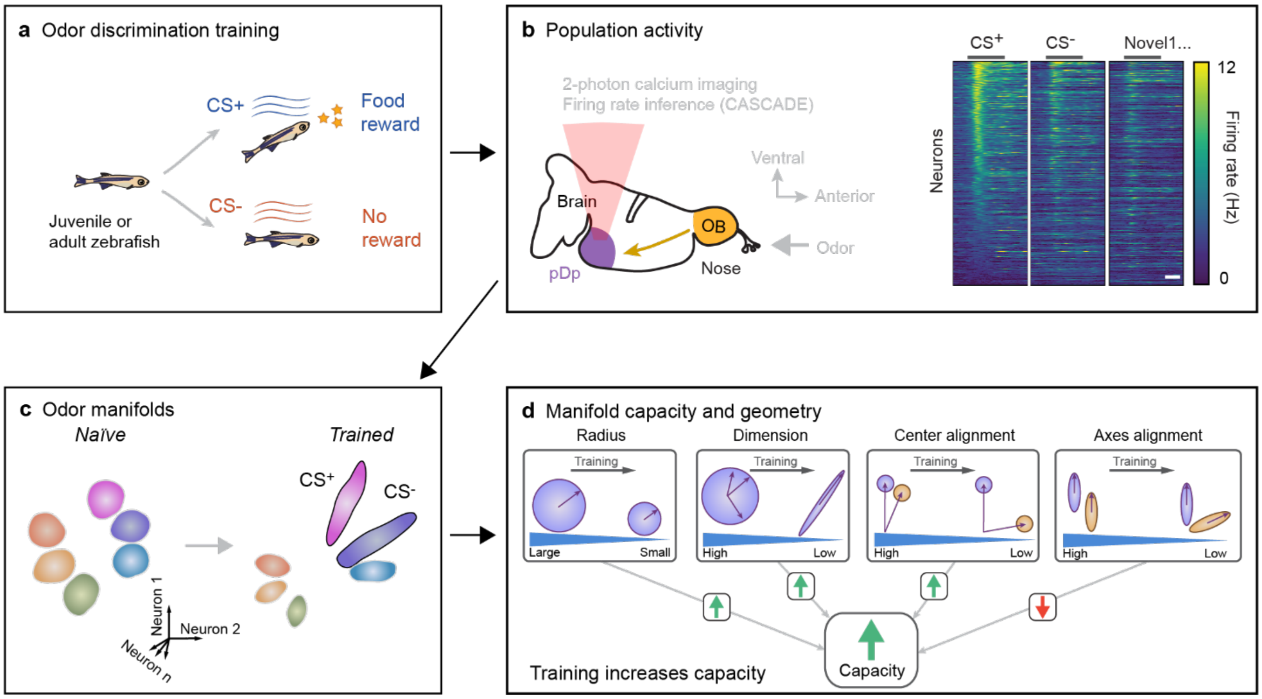


**Supplementary Fig. S1 | Graphical summary of approach and main results.** **a**, Juvenile or adult zebrafish were trained in an odor discrimination task. **b**, Population activity evoked by conditioned ($CS^+$, $CS^-$) and novel odors was measured in telencephalic area pDp, the homolog of piriform cortex, using 2-photon calcium imaging and firing rate inference. **c**, Training enhanced the separation of neural manifolds representing conditioned odors ($CS^+$, $CS^-$) from representations of other odors. **d**, Analyses based on *manifold capacity theory* demonstrated that training enhanced the linear separability, or "untangeledness", of manifolds representing conditioned odors. Increased manifold capacity (separability) could be attributed to changes in multiple geometrical features: (1) a decrease in the effective radius (*manifolds become more "compact"*), (2) a decrease in dimensionality (*manifolds become more "flat"*) and (3) a decrease in center alignment (*manifolds become more decorrelated*). A concomitant decrease in axes alignment (*manifolds become less aligned*) had a negative effect on manifold capacity that was, however, outweighed by changes in the other features. The increase in manifold capacity was correlated to odor discrimination performance across individuals, indicating that geometrical features of representational manifolds are closely related to the behavioral readout of odor representations.

bioRxiv preprint doi: https://doi.org/10.1101/2024.11.17.623906; this version posted November 18, 2024. The copyright holder for this preprint (which was not certified by peer review) is the author/funder, who has granted bioRxiv a license to display the preprint in perpetuity. It is made available under aCC-BY 4.0 International license.

s

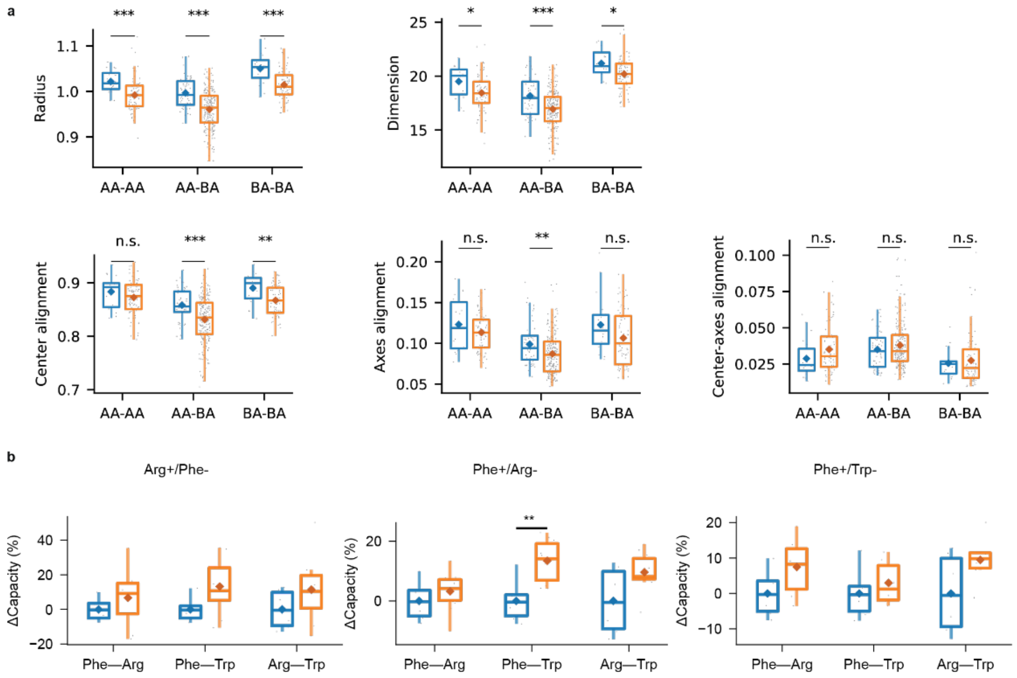


**Supplementary Fig. S2 | Further analysis of geometrical measures of representational manifolds and changes in manifold capacity in juvenile zebrafish**

**a,** Effective geometric measures in different classes of odor pairs. Radius: amino acids vs amino acids (AA-AA) [naïve] 1.02 ± 0.03 (mean ± SD), n = 18 pairs from N = 6 fish, [trained] mean ± SD 0.99 ± 0.04, n = 75 odor pairs from N = 25 fish, Mann–Whitney U test, P = 0.0004. Amino acids vs bile acids (AA-BA) [naïve] 1.00 ± 0.03, n = 54 pairs from N = 6 fish, [trained] 0.96 ± 0.04, n = 225 pairs from N = 25 fish, Mann–Whitney U test, P = 4.2 × $10^{-8}$. Bile acids vs bile acids (BA-BA) [naïve] 1.05 ± 0.03, n = 18 pairs from N = 6 fish, [trained] 1.02 ± 0.03, n = 75 pairs from N = 25 fish, Mann–Whitney U test, P = 0.0002. Dimension: AA-AA [naïve] 19.5 ± 1.5, [trained] 18.4 ± 1.7, P = 0.018. AA-BA [naïve] 18.2 ± 1.7, [trained] 16.9 ± 1.8, P = 5.4 × $10^{-5}$. BA-BA [naïve] 21.2 ± 1.3, [trained] 20.2 ± 1.5, P = 0.013. Center alignment: AA-AA [naïve] 0.88 ± 0.03, [trained] 0.87 ± 0.03, P = 0.226. AA-BA [naïve] 0.86 ± 0.03, [trained] 0.83 ± 0.04, P = 3.2 × $10^{-5}$. BA-BA [naïve] 0.89 ± 0.03, [trained] 0.87 ± 0.03, P = 0.004. Axes alignment: AA-AA [naïve] mean ± SD 0.123 ± 0.032, [trained] 0.113 ± 0.023, P = 0.296. AA-BA [naïve] 0.099 ± 0.026, [trained] 0.087 ± 0.024, Mann–Whitney U test, P = 0.003. BA-BA [naïve] 0.123 ± 0.035, [trained] 0.106 ± 0.034, Mann–Whitney U test, P = 0.103. Center-axes alignment: AA-AA [naïve] 0.029 ± 0.013, [trained] 0.035 ± 0.016, P = 0.079. AA-BA [naïve] 0.035 ± 0.013, [trained] 0.038 ± 0.016, P = 0.299. BA-BA [naïve] 0.026 ± 0.010, [trained] 0.027 ± 0.017, P = 0.687. **b,** Change in capacity in each training group relative to the naïve group for each pair of amino acids. Capacity was consistently higher in trained fish but statistical significance was reached only in one case (Phe-Trp in $Phe^+/Arg^-$ fish; P = 0.002), possibly because the number of datapoints for comparison of single odor pairs in individual training groups is low. See Fig. 7 for statistical comparisons of pooled data.
